# Functional Characterization of Soybean Diacylglycerol Acyltransferase 3 in Yeast and Soybean

**DOI:** 10.3389/fpls.2022.854103

**Published:** 2022-05-25

**Authors:** Jinai Xue, Huiling Gao, Yinghong Xue, Ruixiang Shi, Mengmeng Liu, Lijun Han, Yu Gao, Yali Zhou, Fei Zhang, Haiping Zhang, Xiaoyun Jia, Runzhi Li

**Affiliations:** ^1^College of Agriculture, Institute of Molecular Agriculture and Bioenergy, Shanxi Agricultural University, Taigu, China; ^2^College of Landscape Architecture, Northeast Forestry University, Haerbin, China; ^3^Center for Agricultural Genetic Resources Research, Shanxi Agricultural University (Institute of Crop Germplasm Resources, Shanxi Academy of Agricultural Sciences), Taiyuan, China

**Keywords:** soybean (*Glycine max* (L.) Merr.), diacylglycerol acyltransferase 3 (DGAT3), yeast functional complementation assay, genetic transformation of tobacco (*Nicotiana benthamiana*), fatty acid and TAG biosynthesis

## Abstract

Diacylglycerol acyltransferases (DGAT) function as the key rate-limiting enzymes in *de novo* biosynthesis of triacylglycerol (TAG) by transferring an acyl group from acyl-CoA to *sn*-3 of diacylglycerol (DAG) to form TAG. Here, two members of the *type 3 DGAT* gene family, *GmDGAT3-1* and *GmDGAT3-2*, were identified from the soybean (*Glycine max*) genome. Both of them were predicted to encode soluble cytosolic proteins containing the typical thioredoxin-like ferredoxin domain. Quantitative PCR analysis revealed that *GmDGAT3-2* expression was much higher than *GmDGAT3-1's* in various soybean tissues such as leaves, flowers, and seeds. Functional complementation assay using TAG-deficient yeast (*Saccharomyces cerevisiae*) mutant H1246 demonstrated that GmDGAT3-2 fully restored TAG biosynthesis in the yeast and preferentially incorporated monounsaturated fatty acids (MUFAs), especially oleic acid (C18:1) into TAGs. This substrate specificity was further verified by fatty-acid feeding assays and *in vitro* enzyme activity characterization. Notably, transgenic tobacco (*Nicotiana benthamiana*) data showed that heterogeneous expression of *GmDGAT3-2* resulted in a significant increase in seed oil and C18:1 levels but little change in contents of protein and starch compared to the EV-transformed tobacco plants. Taken together, GmDGAT3-2 displayed a strong enzymatic activity to catalyze TAG assembly with high substrate specificity for MUFAs, particularly C18:1, playing an important role in the cytosolic pathway of TAG synthesis in soybean. The present findings provide a scientific reference for improving oil yield and FA composition in soybean through gene modification, further expanding our knowledge of TAG biosynthesis and its regulatory mechanism in oilseeds.

## Introduction

Soybean (*Glycine max* [L.] Merr.), an important crop in the world, can accumulate 40% protein and 20% oil, respectively, in its seeds (Hartman et al., 2011; Sugiyama et al., 2015). Particularly, soybean oil makes up over half of the global oilseed production, serving as one of the key contributors to human food, biofuel, and oleochemical industries worldwide (Xie et al., 2011; Li et al., 2013). The quality and nutritional value of oils and oil-derived foods depend on their fatty acid profiles. Soybean oil contains about 80% of polyunsaturated fatty acids (PUFAs). The high amount of PUFAs make soybean oil easily oxidized by lipoxygenase, and then generated a beany flavor (Amin et al., 2019). Vegetable oils with a high level of monounsaturated fatty acids (MUFAs) are much better suited for human health and industrial processing and utilization (Xu and Gai, 2003; Wang et al., 2012). Therefore, genetic improvement of oil yield and fatty acid composition is the main research field in soybean and other oilseeds despite the molecular mechanism underlying fatty acid/oil biosynthesis and regulation remains to be elucidated in detail. The majority of seed oils are stored in form of triacylglycerol (TAG), which is formed by esterification of three fatty acids (FAs) located, respectively, at the positions *sn-1, sn-2*, and *sn-3* of the glycerol backbone (Browse et al., 1986; Zweytick et al., 2000; Yu and Ginsberg, 2009). TAGs synthesized in plant seeds provide the major source of nutrients for seed germination and early seedling growth (Carrera et al., 2007; Bassel et al., 2008; Theodoulou and Eastmond, 2012; Yang and Benning, 2018). TAG biosynthesis takes place in the endoplasmic reticulum (ER), which is consecutively catalyzed by three different acyltransferases using acyl-CoA in the cytoplasm as the fatty acyl donor. The first enzyme in this pathway, glycerol-3-phosphate acyltransferase (GPAT; EC 2.3.1.15), catalyzes the formation of lysophosphatidic acid (LPA) by transferring the fatty acyl of acyl-CoA to the *sn-1* position of glycerol-3-phosphate (G3P). Then, lysophosphatidic acid acyltransferase (LPAAT; EC 2.3.1.51) transfers the fatty acyl of acyl-CoA into the *sn-2* position of LPA to form phosphatidic acid (PA). Subsequently, under the action of phosphatidic acid phosphatase (PAP; EC 3.1.3.4), the phosphate radical at the *sn-3* position is removed to form diacylglycerol (DAG). Finally, diacylglycerol acyltransferase (DGAT, EC3.2.1.20) transfers the fatty acyl of acyl-CoA into the *sn-3* position of DAG to form triacylglycerol (Durrett et al., 2010; Pan et al., 2015). TAGs then were stored in an oil body.

Diacylglycerol acyltransferase is the final and rate-limiting enzyme in the TAG biosynthesis pathway, which determines the carbon flow toward TAGs (Liu et al., 2012; Caldo et al., 2015). Currently, four types of DGATs have been characterized, including DGAT1, DGAT2, DGAT3, and Wax Ester Synthase (WS)/DGAT. The phylogenetic and evolutionary analysis demonstrated that these DGATs not only share little sequence similarity with each other but also cluster into four independent groups. Although they may originate from different ancestors, four types of DGATs exhibit similar catalytic activity in TAG assembly by transferring a fatty acyl to DAG (Turchetto-Zolet et al., 2016). DGAT1 belongs to the membrane-bound O-acyltransferase (MBOAT) family and shares high homology with the acyl-CoA cholesterol acyltransferase (ACAT) family. *DGAT1* gene was firstly cloned in mice (*Mus musculus*) (Cases et al., 1998), followed by the identification of numerous *DGAT1s* from many organisms. *DGAT2* gene encoding another enzyme protein with DGAT activity was examined in various species such as fungus *Mortierella ramanniana* (Lardizabal et al., 2008) and yeast as well as higher plants. DGAT1 and DGAT2 display differences in their intracellular localization, substrate specificity, and gene expression patterns (Shockey et al., 2006; Li et al., 2010). The bifunctional WS/DGAT was identified in the bacterium *Acinetobacter calcoaceticus* (Kalscheuer and Steinbuchel, 2003) and in higher plant Arabidopsis (*Arabidopsis thaliana*). The loss of function mutant *WSD1* (*WS/DGAT-like1*) resulted in the wax deficiency in the stem of Arabidopsis (Li et al., 2008). Unlike DGAT1 and DGAT2 present as membrane-bound ER-localized proteins, DGAT3 is a soluble cytosolic protein, which was initially identified in developing peanut (*Arachis hypogaea*) cotyledons (Saha et al., 2006), and recently in Arabidopsis (Hernandez et al., 2012) and yeast (*Saccharomyces cerevisiae*) (Rani et al., 2013). Arabidopsis DGAT3 protein contains a [2Fe-2S] cluster involved in TAG biosynthesis, showing substrate specificity for 18:3 and 18:2 acyl chains (Hernandez et al., 2012).

Compared to DGAT1 and DGAT2 which are well-characterized in many plant species, DGAT3 has been examined only in a few species. Most of the functions of DGAT3 members await investigation extensively. Here, we identified the two soybean genes encoding DGAT3, namely *GmDGAT3-1* and *GmDGAT3-2*. Expression profiling in various tissues by qRT-PCR revealed that *GmDGAT3-2* was largely expressed in these tissues tested, with the highest level in developing seeds. Furthermore, the GmDGAT3-2 enzyme was functionally characterized by both heterologous overexpression in a yeast TAG-defective yeast mutant H1246 and transient expression in *Nicotiana benthamiana* leaves, followed by tobacco genetic transformation. This study provides new insights into the mechanism by which soybean *DGAT3-2* gene functions importantly for TAG biosynthesis and regulation.

## Materials and Methods

### Plant Materials

Soybean variety “Jack” was used in this study. Soybeans were planted in the fields of experimental stations at Shanxi Agricultural University, Shanxi, China. Wild-type tobacco (*Nicotiana benthamiana*) and the transgenic tobacco lines were grown in the nutritious soil with a photoperiod of 16 h light:8 h dark in a greenhouse.

### Strains of Yeasts and Bacteria

Yeast (*Saccharomyces cerevisiae*) wild-type strain INVSc1 was bought from Chengdu Transvector Biotechnology Company. The TAG-deficient yeast strain H1246 (*MAT*α *are1-*Δ*::HIS3 are2-*Δ*::LEU2 dga1-*Δ*::KanMX4 Iro1-*Δ*::TRP1 ADE2 ura3*) (Sandager et al., 2002) was a kind gift from Mr. Tailong Tan from Hunan Agricultural University. Agrobacterium (*Agrobacterium tumefaciens*) strain GV3101 and *Escherichia coli* DH5α were all maintained at the Institute of Molecular Agriculture and Bioenergy of Shanxi Agricultural University.

### Cloning of *GmDGAT3-2* Gene and Plasmid Construction

Among the transcriptome data derived from the developing seed of soybean Jack, two cDNAs were annotated as *GmDGAT3*, and further named as *GmDGAT3*-*1* and *GmDGAT3-2*, respectively, according to the genome-wide characterization of soybean GmDGAT3 sequences (see below). The cDNA sequence of *GmDGAT3-2* was used as the template to design the gene-specific primers (Supplementary Table 2), which then were used for RT-PCR amplification of the encoding sequence (ORF) of *GmDGAT3-2* from soybean developing seeds of Jack. The resulted PCR product (1,014 bp) was ligated into the pEAST Blunt Zero Cloning Vector, and the clone vectors were further transformed into *Escherichia coli* DH5α. The positive bacteria clone was selected for further sequencing analysis.

For yeast expression vector construction, the *GmDGAT3-2* coding sequence was cloned into the pYES2 vector under the control of the galactose-inducible GAL1 promoter. For the construction of the plant expression vector, the *GmDGAT3-2* coding sequence was inserted into the pCAMBIA1303 vector under the control of the cauliflower mosaic virus (CaMV) 35S promoter. The vector of the 35S::GmDGAT3-2-GFP fusion protein was also constructed for subcellular localization of GmDGAT3-2 protein. Both double digestions and sequencing were employed to verify the positive recombinant vectors. Supplementary Table 1 provides a list of all primers used for the construction of the expression vectors.

### Bioinformatic Analysis of Soybean GmDGAT3 Proteins

The AhDGAT3-1 (AAX62735.1) sequences from peanut (*Arachis hypogeal*) were used as a query for Basic Local Alignment Sequence Tool for Protein (BLASTP), and two candidate GmDGAT3s were identified from soybean genome in Phytozome version 12.1 (https://phytozome.jgi.doe.gov/pz/portal.html#!info?alias=Org_Gmax). These two soybean *GmDGAT3* genes were named *GmDGAT3-1* (Glyma.13G118300.1) and *GmDGAT3-2* (Glyma.17G041600.1). A total of 20 DGAT sequences were downloaded from NCBI and Phyzome databases for 9 plant species including *A. thaliana, A. hypogaea, B. napus, C. sativa, C. rubella, E. salsugineum, G. max, P. trichocarpa*, and *V. radiata*. Supplementary Table 1 presented the accession numbers of these 20 *DGAT* genes including *DGAT1s, DGAT2s*, and *DGAT3s*. The 20 DGATs were used for phylogenetic tree analysis.

The online bioinformatics tool EXPASY (http://www.expasy.org) was employed to predict the basic physical and chemical properties of the GmDGAT3 protein family. The putative subcellular localization of GmDGAT3 proteins was predicted with the online tool TargetP (http://www.cbs.dtu.dk/services/TargetP/). The transmembrane regions in GmDGAT3 proteins were detected with the online tool TMHMM Server (version 2) (http://www.cbs.dtu.dk/services/TMHMM/). The secondary structure of GmDGAT3 and peanut AhDGAT3-1 protein was deduced using the online tool SOPMA (https://npsa-prabi.ibcp.fr/cgi-bin/npsa_automat.pl?page=npsa_sopma.html). The conserved domains of GmDGAT3 protein were identified by the SMART database (http://smart.emblheidelberg.de/) and Conserved Domain Database (CDD, http://www.ncbi.nlm.nih.gov/Structure/cdd/wrpsb.cgi) at the National Center for Biotechnology Information (NCBI, http://www.ncbi.nlm.nih.gov/). The three-dimensional structure of the proteins encoded by the GmDGAT3 genes was characterized using SWISS-MODEL (https://swissmodel.expasy.org/interactive). The online tool PRALINE (http://www.ibi.vu.nl/programs/pralinewww/) was taken to conduct an alignment analysis of soybean GmDGAT3 and the related proteins. A phylogenetic tree of GmDGAT3 and other DGAT3 proteins was constructed by the neighbor-joining method in the MEGA 6.0 (see Supplementary Table 2 for protein information derived from 9 different plants species).

### qRT-PCR and Semi-quantitative RT-PCR

The relative mRNA levels were determined by both semi-quantitative RT-PCR and qRT-PCR. The semi-quantitative RT-PCR was conducted in a 10 μL reaction system consisting of 0.2 μL cDNA, 5 μL 2 × Taq PCR MasterMix (Applied Biological Materials Inc., USA), 0.8 μL gene-specific primers (Table 1), and 4 μL ddH_2_O. The PCR reaction was run with an initial denaturation at 94°C for 10 min, followed by 25 cycles of 94°C for 30 s, 55°C for 30 s, 72°C for 1 min, and a final extension at 72°C for 10 min.

**Table 1:** Fatty acid profiles of H1246 yeast expressing *GmDGAT3-2* and the controls cultured in the medium supplemented with exogenous fatty acids.

	**C16:0**	**C16:1**	**C18:0**	**C18:1**	**C18:2**	**C18:3**
No addition of exogenous FA						
*ScDGA1*	8.61 ± 0.89	33.74 ± 0.74	9.74 ± 0.2	41.31 ± 1.02		
*GmDGAT3-2*	6.34 ± 0.76	37.03 ± 0.34	6.66 ± 0.57	47.83 ± 0.25		
C16:0 addition						
*ScDGA1*	11.02 ± 0.45	32.19 ± 0.97	9.83 ± 0.34	41.82 ± 0.93		
*GmDGAT3-2*	9.12 ± 0.56	34.56 ± 0.63	7.42 ± 0.29	43.76 ± 0.58		
C16:1 addition						
*ScDGA1*	9.29 ± 0.75	35.12 ± 0.59	9.72 ± 0.28	40.73 ± 0.73		
*GmDGAT3-2*	5.89 ± 1.03	39.26 ± 0.76^*^	7.18 ± 0.93	45.53 ± 1.12		
C18:1 addition						
*ScDGA1*	9.66 ± 0.81	32.99 ± 1.09	9.31 ± 0.37	44.71 ± 0.84		
*GmDGAT3-2*	4.14 ± 1.26^*^	33.47 ± 0.88	4.95 ± 0.87^*^	52.7 ± 0.43^**^		
C18:2 addition						
*ScDGA1*	8.12 ± 1.32	31.85 ± 0.82	9.41 ± 0.79	39.97 ± 0.58	4.68 ± 0.46	
*GmDGAT3-2*	5.36 ± 0.54^*^	34.67 ± 0.66	6.38 ± 0.71^*^	44.65 ± 0.39	5.87 ± 0.65	
C18:3 addition						
*ScDGA1*	8.84 ± 0.77	32.18 ± 1.03	9.02 ± 0.53	40.06 ± 0.48		4.76 ± 0.61
*GmDGAT3-2*	5.63 ± 0.37^*^	35.65 ± 0.20	5.81 ± 1.20^*^	44.71 ± 0.47		5.09 ± 1.01

*Values are mean ± SE (n = 3). Asterisks indicate significant difference from the wild type according to t-test at*

** *P < 0.01, and*

* *P < 0.05, respectively*.

qRT-PCR was performed using EvaGreen 2 × qPCR MasterMix (Applied Biological Materials Inc., USA). The 10 μL reaction system included 0.5 μL cDNA, 5 μL EvaGreen 2 × qPCR MasterMix, 0.6 μL gene-specific primers (Table 1), and 3.9 μL ddH_2_O. The PCR reaction was run with denaturation at 95°C for 10 min, followed by 40 cycles of 95°C for 15 s, 55°C for 1 min, 72°C for 1 min, and a final extension at 72°C for 10 min. A 2–ΔΔcq calculation was used to determine the relative mRNA levels normalized to the internal control *GmActin* gene.

### Yeast Transformation and Complementation Assay

The pYES2-GmDGAT3-2 plasmid was transformed into yeast mutant H1246, which is deficient in TAG biosynthesis. The H1246 transferred with yeast DGAT2 (ScDGA1) was used as the positive control, and the yeast H1246 bearing the empty pYES2 vector was used as a negative control. The transformants were screened on synthetic minimal defined medium lacking uracil (SC-Ura) with the addition of 2% (w/v) glucose. The positive colonies were cultured in liquid SC medium-Ura with 2% (w/v) glucose at 30°C. After that, the yeast cultures were diluted to OD600 = 0.4 in liquid SC medium-Ura with 2% (w/v) galactose for transgene expression induction at 30°C. The yeast cells were collected by centrifugation (8,000 rpm, 4°C, 10 min), and then, the samples were washed three times with distilled water for late use in lipid analysis.

For yeast feeding assays, palmitic acid (16:0), palmitoleic acid (16:1), oleic acid (18:1), linoleic acid (18:2), and α-linolenic acid (18:3) were first dissolved in Tween 80, respectively, and then added into the medium with a final concentration of 1 mM, respectively, to the culture medium of yeast H1246 overexpressing *GmDGAT3-2* or yeast *ScDGA1* (as the control) (Zhang et al., 2017). The cells were harvested by centrifugation (8,000 g for 5 min), washed three times with double-distilled water, and subjected to lipid extraction and then for TAG analysis.

### GmDGAT3 Protein Expression, Purification and *in vitro* Enzyme Activity Assays

To investigate enzyme activity and acyl-CoA substrate specificity of GmDGAT3-2, an *in vitro* assay was performed. ORF of *GmDGAT3-2* gene was inserted to pET 28 a^+^ vector between *EcoR*I and *Xho*I to form a prokaryotic expression vector pET 28 a^+^-*GmDGAT3-2*. The plasmid was introduced into *E. coli* BL21 (DE3) competent cells. Transformed *E. coli* cells were grown at 37°C in an LB medium containing 30 μg/mL kanamycin until the OD600 reached 0.3. Afterward, the temperature was lowered from 37 to 18°C and isopropyl β-Dthiogalactoside (IPTG) was added to a final concentration of 0.1 mM. After 24 h culture under this condition, the cells were harvested by centrifugation (6,000 × *g* at 4°C for 10 min) and were re-suspended in 20 mM sodium phosphate buffer (5 mM imidazole, 0.2 mg/mL lysozyme, 20 μg/mL DNase, and 1 mM MgCl_2_) and then disrupted by ultra-sonication. The supernatant containing soluble proteins was obtained by centrifugation (13,100 × *g* at 4°C for 15 min). The target proteins were collected by loading the supernatant into a Ni-Sepharose 6 FF column (GE Healthcare, Piscataway, NJ, USA). To establish an *in vitro* reaction system, 18:1n9/16:0 DAG (sn-1,2-diacylglycerol) was used as an acyl acceptor, and16:0-CoA, 16:1-CoA, 18:1-CoA, 18:2-CoA, and 18:3-CoA were, respectively, added into the reaction buffer solution, followed by adding the extracted enzyme protein. After the reaction solution was incubated for 1 h, TAG content in the solution was detected to determine the activity of GmDGAT3-2 in the presence of each acyl-CoA in the medium.

### Nile Red Staining of Yeast Cells

One mL of yeast cells were collected and then diluted to OD600 = 0.3 with distilled water. Hundred micro liter of yeast cells were stained with 1 mL staining solution consisting of 10 μL of Nile Red (5 mg·mL^−1^), 50 μL of dimethyl sulfoxide (DMSO), and 850 μL distilled water for 1 min, followed by fluorescence microscopy observation. The stained yeast cells were observed and recorded by fluorescence microscope on a PH50TV (Shanghai Rongjida Test Equipment Co., Ltd. shanghai, China) using a 340 nm excitation wavelength.

### Tobacco Transient Expression and Stable Transformation

The plant expression vector pCAMBIA1303-*GmDGAT3-2* was introduced into *Agrobacterium* strain GV3101 by the freeze-thaw method. Agrobacterium colonies were grown in LB medium at 28°C overnight, and then cell pellets were collected by centrifugation of cell cultures. The pellet was resuspended in infiltration buffer (10 mmol·L^−1^ MES buffer with 100 μmol·L^−1^ acetosyringone and 10 mmol·L^−1^ MgSO_4_). The resuspended cells were infiltrated on one side of the leaf blade of 6-week-old *Nicotiana benthamiana* leaves until the infiltrated areas spread to a region of 1 cm diameter. On the other side of the main leaf vein, the cell suspensions of wild-type *Agrobacterium* containing the empty vector were infiltrated as the negative control. After 3 days of infection, the infected leaf samples were harvested for RNA extraction and expression analysis of the target gene. After 6 days of infection, the infected *N. benthamiana* leaves were collected, and then freeze-dried for extraction of total lipids and fatty acid profiling.

The *GmDGAT3-2* gene expression vector was transformed into leaf discs of 30-day sterile tobacco seedlings using *Agrobacterium*-mediated transformation. Transgenic shoots were selected on kanamycin (50 mg/ml) and were rooted on 1/2 MS medium containing kanamycin (50 mg/ml). The rooted shoots were transferred to the potting mixture and hardened plants were established in a growth chamber. The homozygous transgenic plants were obtained by screening positive transgenic tobacco plants. The transgenic plants were identified by PCR using genomic DNA as the template. *GmDGAT3-2* expression was examined by RT-PCR using RNA as the template prepared from the transgenic tobacco plants. Analysis of oil content and fatty acid profiles in the transgenic tobacco seeds were conducted using the methods described above.

### Subcellular Localization of GmDGAT3-2 Protein

A pCAMBIA1300-GmDGAT3-2-GFP fusion plasmid was constructed. This plasmid was transformed into competent cells of *Agrobacterium* strain GV3101 by the freeze-thaw method. Then, the method of tobacco transient expression was as same as the above description. After normal culture for 3 days, the tobacco leaf () was observed with confocal laser microscopy and leaf cell images were taken.

### Total Lipid Exaction and Fatty Acid Profiles by GC Analysis

Total lipids were extracted, respectively, using the chloroform-methanol method from the transgenic H1246 yeast cells (transformed with empty pYES2, pYES2-*GmDGAT3-2*, and pYES2-*ScDGA1*, respectively) and also from the target gene transiently-expressed *N. benthamiana* leaves and the transgenic tobacco seeds (El Tahchy et al., 2017). The total lipid content was obtained by weighing the change in the weight of the glass tube. Then, the total lipid samples were finally dissolved in n-hexane. For yeast feeding assays, we specifically analyzed the TAG contents and fatty acid composition. TAGs were separated from lipids dissolved in n-hexane by thin-layer chromatography (TLC) using a solvent mixture of n-hexane/diethyl ether/acetic acid (80:20:2, v/v/v) (Browse et al., 1986; Wang and Benning, 2011). TAG spots were scraped from the TLC plates and then used to prepare FAMEs for further analysis by gas chromatography (GC). The TAG content was obtained by weighing the change of the glass tube.

Fatty acid methyl esters (FAMEs) were prepared from TAG spots after purification. Briefly, 500 μL of 2.5% concentrated sulfuric acid-methanol was added to the lipid sample for methyl esterification in a water bath at 80°C for 2 h. After drying under nitrogen flow, the FAME sample was dissolved in n-hexane before being transferred to a GC bottle. Each sample was conducted in triplicate. FAMEs were analyzed with an Agilent 7890B gas chromatograph equipped with a hydrogen ion flame (FID) detector and an HP-88 chromatographic column (100 m × 0.25 mm, 0.25 μm) (Agilent Technologies Inc., USA).

### Measurement of Protein and Starch Contents in the Transgenic Tobacco Leaves

Protein content was measured using the Bradford protein content assay kit (Sangon Biotech Co., Ltd. Shanghai, China). Starch content was tested by the anthrone colorimetric method. The experiment was carried out at least three biological repeats.

### Statistical Analysis of Experimental Data

All experiments were carried out at least in triplicate. The data was analyzed using IBS SPSS Statistics software version 22 (IBM Corp, Armonk, NY, USA). Duncan's test was employed to detect the statistical significance (*P* < 0.05) when comparing differences between two groups.

## Results

### Identification and Sequence Analysis of DGAT3 Family Members From Soybean

**Figure 1:**
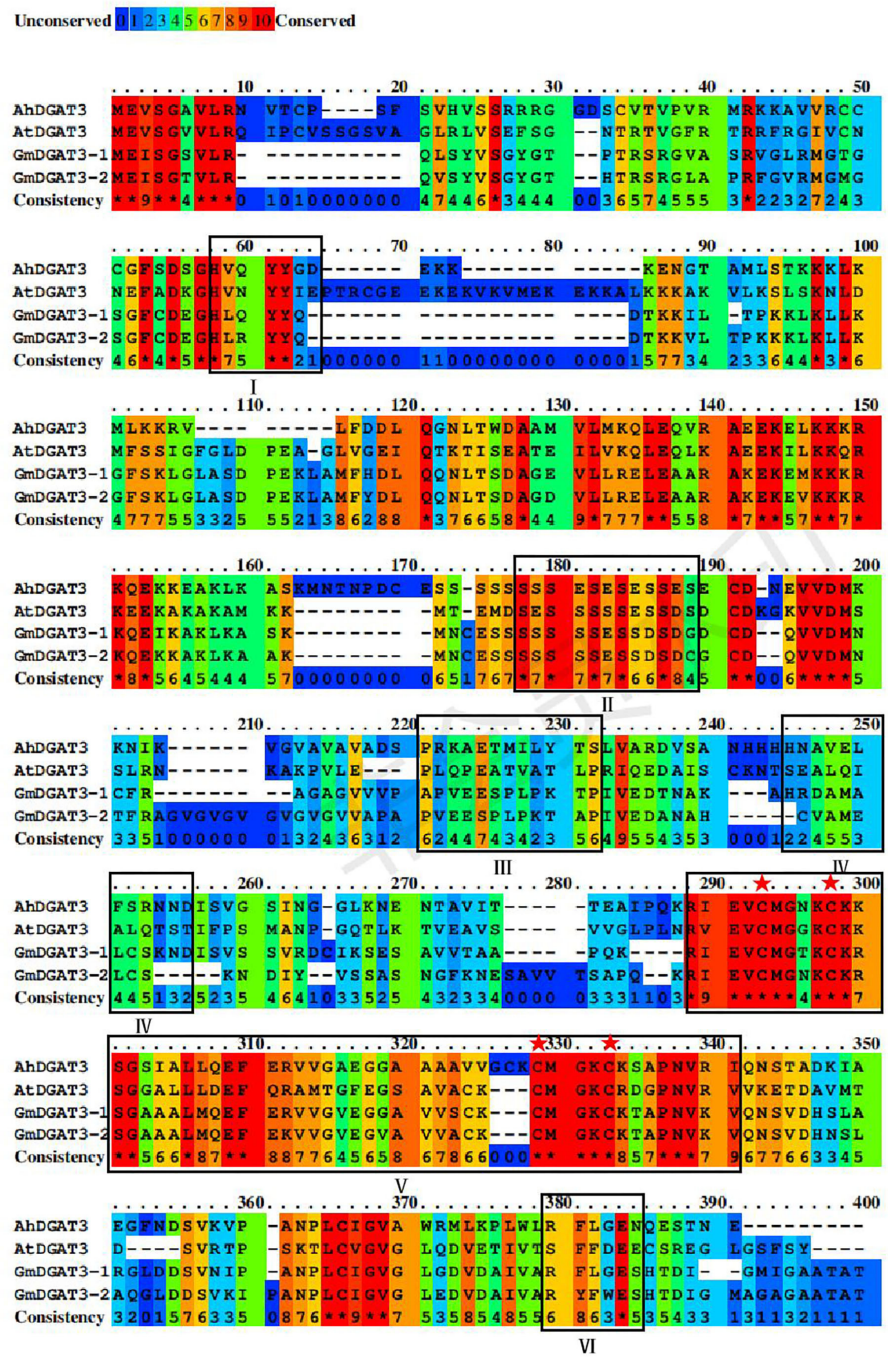
Sequence alignment of GmDGAT3, AhDGAT3-1, and AtDGAT3 proteins. The conserved amino acids were denoted by different colors, and residues in red represent 100% conservation of all sequences. The conserved domains were framed and marked by I–VI. The potential DGAT motifs were marked as I and IV. DGAT catalytic motif was labeled as VI. A putative thiolase acyltransferase intermediate signature was labeled as II. A putative phosphorylation conservation site of Tyr (tyrosine) kinase was designated as III. And the thioredoxin-like ferredoxin domain was a symbol as V with its conserved cysteine residues was highlighted with red asterisks.

Based on a BLAST search of the soybean genome using sequences of the well-studied peanut AhDGAT3-1 (AAX62735.1) as a query, two candidate soybean *DGAT3* genes were identified namely *GmDGAT3-1* (Glyma.13G118300.1) and *GmDGAT3-2* (Glyma.17G041600.1) (Supplementary Table 2). Analysis by SMART and CDD online tools revealed that GmDGAT3 proteins encoded by these two genes contained a thioredoxin-like ferredoxin (TRX-like Fd) domain harboring four conserved cysteines possibly involved in the binding of a [2Fe-2S] cluster (Turchetto-Zolet et al., 2016; Aymé et al., 2018; Rosli et al., 2018) (Figure 1).

To explore the functions of these GmDGAT3 proteins, their physio-chemical properties were examined using bioinformatics tools. As shown in Supplementary Table 3, GmDGAT3-1 and GmDGAT3-2 were 327 and 338 amino acids in length, respectively, which is slightly shorter than peanut DGAT3 protein consisting of 345 amino acids. The molecular weights were predicted as 34.7 and 35.9 kDa for GmDGAT3-1 and GmDGAT3-2, respectively. Their theoretical isoelectric point (pI) was 8.52 (GmDGAT3-1) and 8.61 (GmDGAT3-2), indicating that both proteins are basic. GmDGAT3 proteins were also hydrophilic and unstable, indicated by their grand average of hydropathicity index (GRAVY) and instability index.

GmDGAT3 proteins were detected to have very similar physicochemical features to peanut AhDGAT3-1. The subcellular localization prediction by the TargetP tool indicated that GmDGAT3 proteins were most likely to localize in the cytoplasm. No transmembrane regions were detected for GmDGAT3 proteins by TMHMM Server (Supplementary Figure 1), indicating that two GmDGAT3s were soluble proteins located in the cytoplasm. The 3D structures of GmDGAT3 proteins were predicted by SWISS (Supplementary Figure 2). Each of those DGAT3 proteins was uniformly symmetrical, consisting of two subunits.

Furthermore, a subcellular localization experiment using transient expressing the GmDGAT3-2-GFP fusion protein in the tobacco showed that GmDGAT3-2 was localized in the cytoplasm (Supplementary Figure 3). This result is consistent with the soluble nature and no transmembrane region of GmDGAT3 predicted by bioinformatics tools.

As shown in Figure 1, GmDGAT3-1 and GmDGAT3-2 had 82% sequence identity, and both shared 45 and 43% sequence similarity with AhDGAT3-1, respectively. The conserved residues are scattered in the N-terminal and C-terminal regions of GmDGAT3s. Potential DGAT motifs labeled as I and IV were identified, as reported for members of the acyltransferase family. An alignment with the known AhDGAT3 also confirmed that the presence of the same DGAT catalytic motif was in GmDGAT3s labeled as VI. The motif labeled as V is the most conserved among DGAT3 proteins, harboring a domain typical of the thioredoxin-like [2Fe-2S] ferredoxin family (cd02980 from NCBI's conserved domain database, Marchler-Bauer et al., 2015) with features only found in homologs to this class of ferredoxins (Aymé et al., 2018). The conserved cysteines are involved in the binding of a [2Fe-2S] cluster in members of the thioredoxin-like ferredoxin family (Yeh et al., 2000; Nouailler et al., 2006). The motif labeled as II and III is, respectively, a putative thiolase acyltransferase intermediate signature and a putative phosphorylation site by a Tyr kinase (Chi et al., 2014). These predicted functional motifs in GmDGAT3-1 and GmDGAT3-2 may therefore indicate their function as bona fide DGAT enzymes in lipid biosynthesis and metabolism.

**Figure 2:**
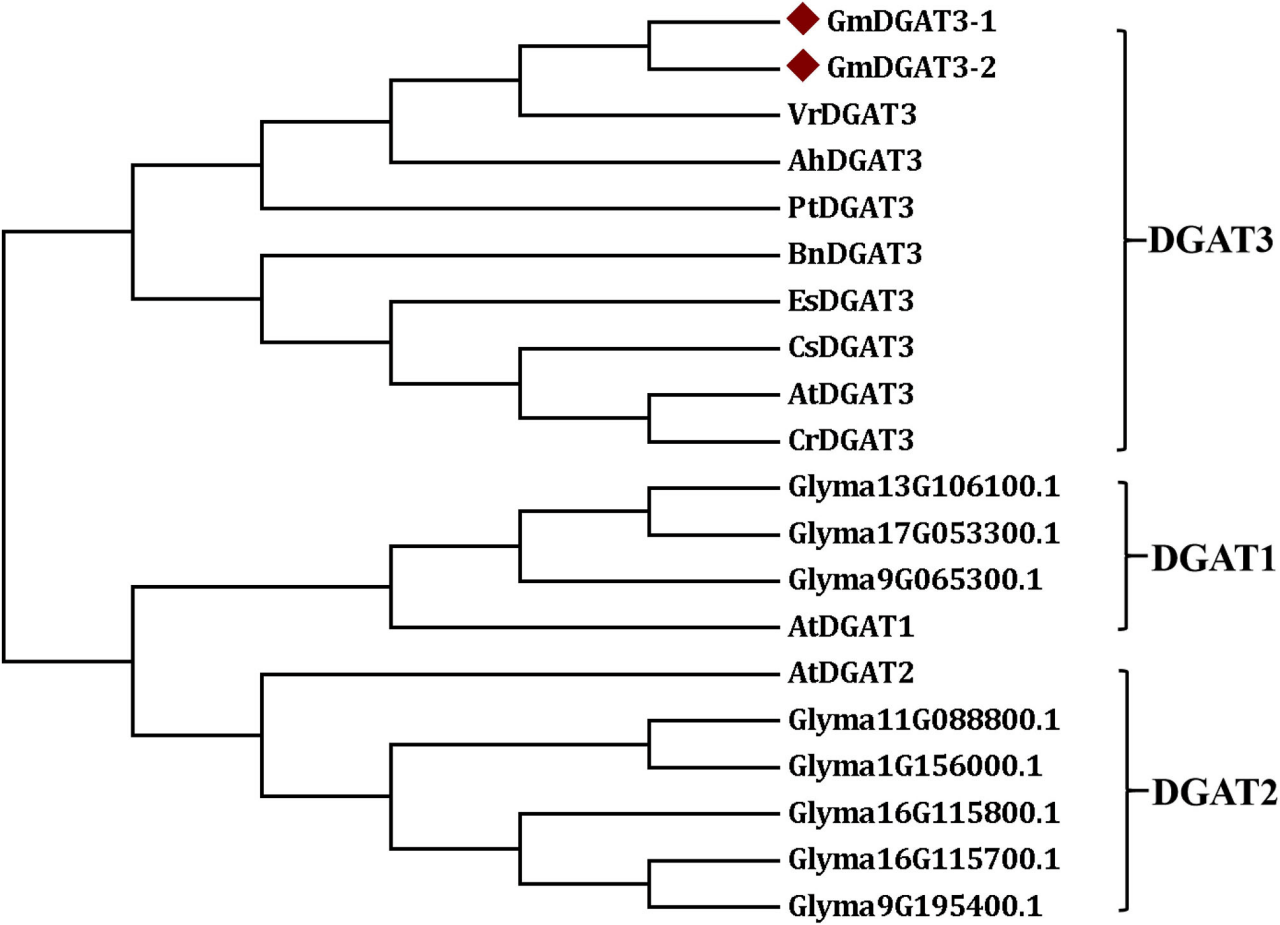
Phylogenetic tree of soybean GmDGAT3s and other plant DGAT3s. The phylogenetic tree was constructed by the Neighbor-Joining method in MEGA6.0. The little red triangle represented soybean GmDGAT3-1 and GmDGAT3-2, respectively. NCBI accession numbers of each DGAT protein were shown in Supplementary Table 1. Gm/Glyma, *Glycine max*; At, *Arabidopsis thaliana*; Ah, *Arachis hypogaea*; Bn, Brassica napus; Cs, *Camelina sativa*; Cr, *Capsella rubella*; Es, *Eutrema salsugineum*; Pt, *Populus trichocarpa*; Vr, *Vigna radiate*.

The sequences of Arabidopsis AtDGAT1, AtDGAT2, and AtDGAT3 were also used as queries to the BLAST soybean genome, respectively. A number of the soybean orthologous proteins were identified, including two DGAT3 (GmDGAT3-1 and GmDGAT3-2), and three DGAT1s (Glyma13G106100.1, Glyma17G053300.1, and Glyma9G065300.1), and five DGAT2s (Glyma9G195400.1, Glyma16G115800.1, Glyma16G115700.1, Glyma1G156000.1, and Glyma11G088800.1). These DGATs and several known DGAT3s from other plant species (Supplementary Table 1), were used to construct the phylogenetic tree by MEGA 7 for further analysis. As shown in Figure 2, all DGAT3s were clustered into one group, which is different from the DGAT1 and DGAT2 groups. In the DGAT3 group, the two GmDGAT3 proteins clustered more closely with the DGAT3s from mung bean (*Vigna radiata*), peanut (*Arachis hypogaea*), and black cottonwood (*Populus trichocarpa*) rather than Brassica BnDGAT3 and other DGAT3s within the same subgroups, indicating that GmDGAT3 may share a common origin with other legume DGAT3s in evolution. Like other legume DGAT3s (e.g., peanut DGAT3) tested, GmDGAT3-1 and GmDGAT3-2 may function importantly in the cytosolic pathway of TAG synthesis in soybean.

### Expression Profiles of *GmDGAT3s* in Various Soybean Tissues

**Figure 3:**
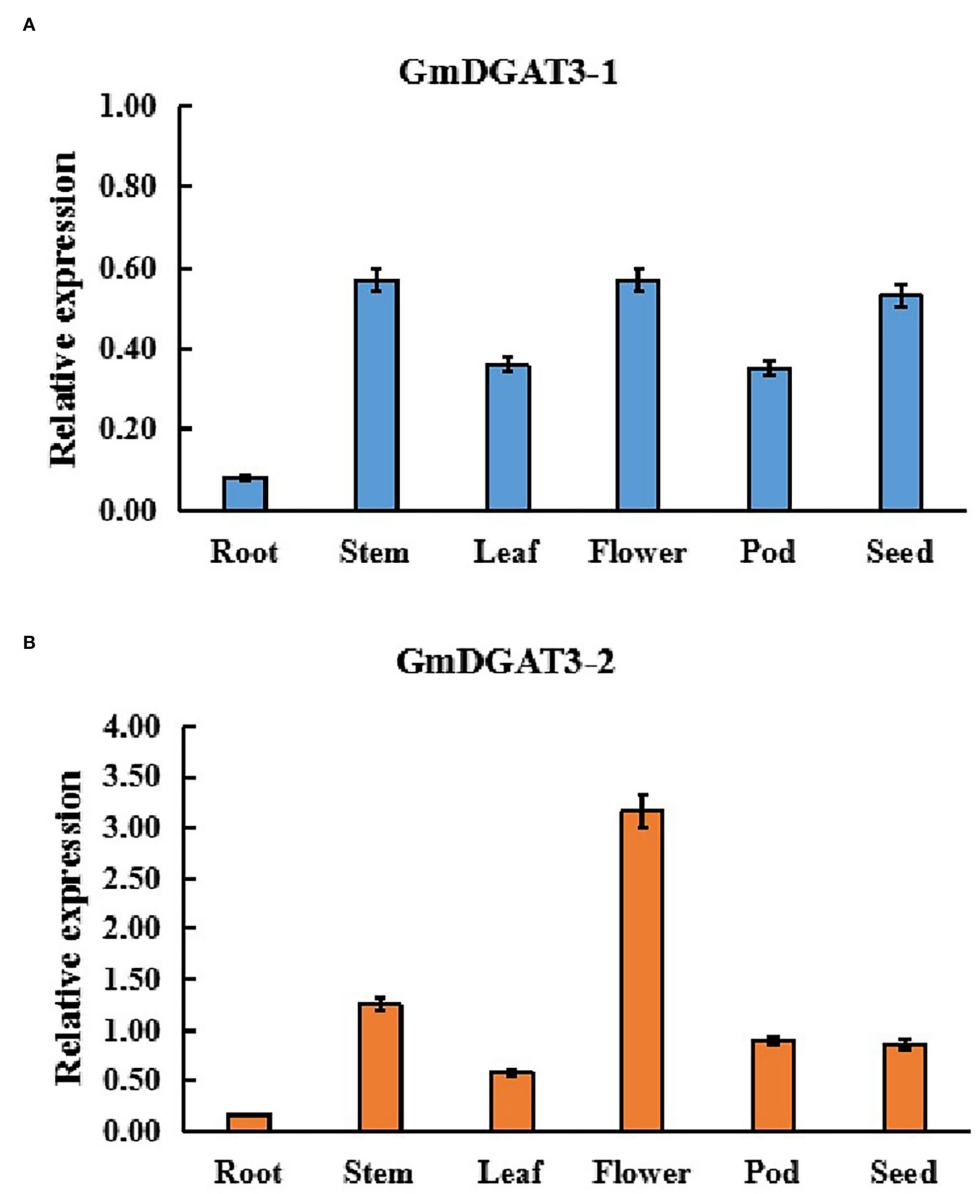
Expression patterns of GmDGAT3-1 **(A)** and GmDGAT3-2 **(B)** in various soybean tissues. Pod: 45 DAF seed pod; Seed: 45 DAF seeds. The days after flowering (DAF) represent each developmental stage of soybean seeds. Data bars represent the mean ± STD level of relative transcript abundance from three biological experiments.

In order to determine the function of GmDGAT3 family members in oil accumulation in soybean, the expression profiles of *GmDGAT3* genes were examined using qRT-PCR in different tissues, including roots, stems, leaves, flowers, seedpods at 45 d and seeds at 45 d (45 days after flowering). Two *GmDGAT3* genes were expressed in various tissues but with distinct patterns (Figure 3). *GmDGAT3-2* expression was stronger than *GmDGAT3-1's* in all tissues tested, with the highest expression in flowers and high expression in stems and seeds (45 d).

**Figure 4:**
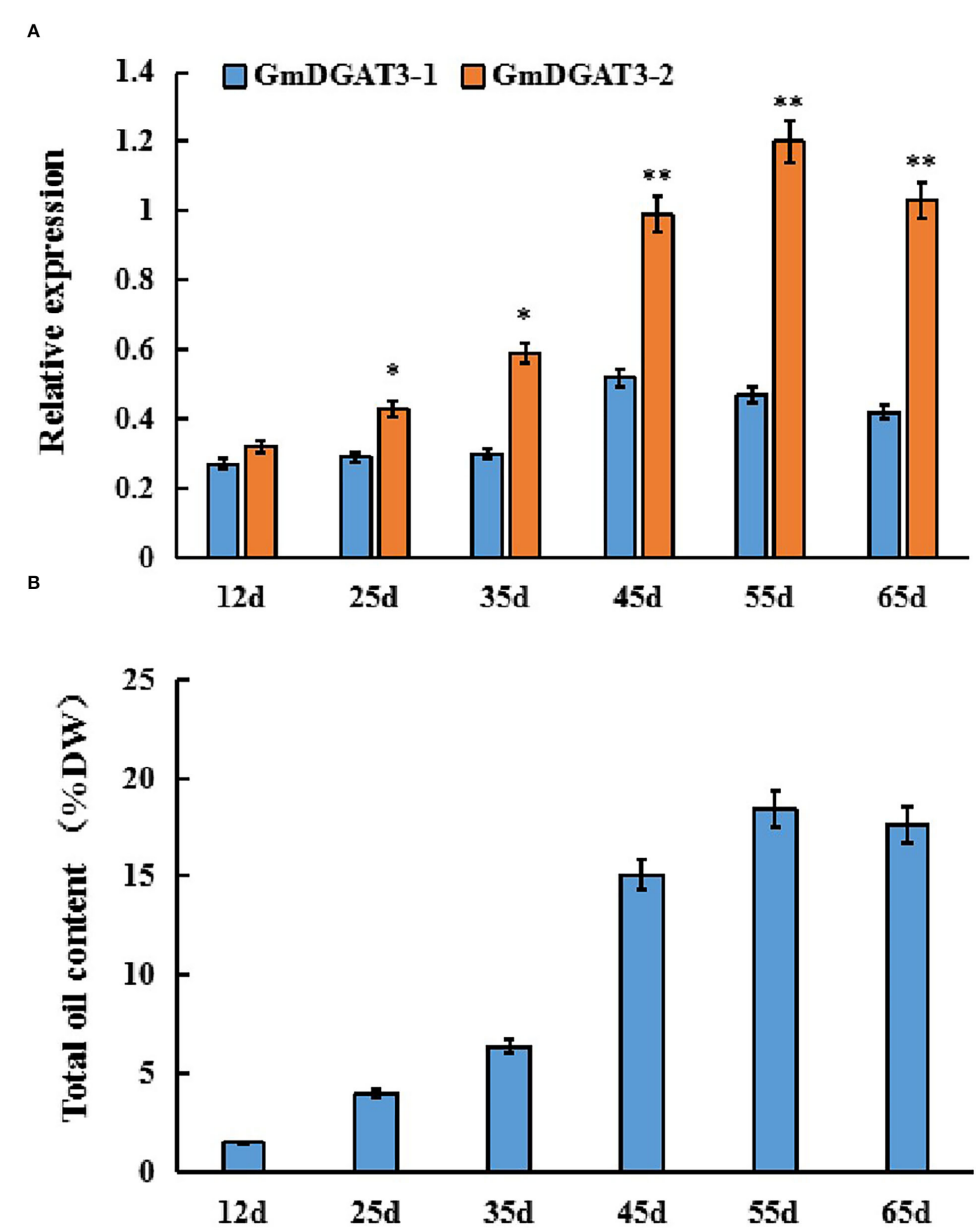
Expression patterns of *GmDGAT3* **(A)** and total oil content **(B)** during soybean seed development. The days after flowering (DAF) represent each developmental stage of soybean seeds. Data bars represent the mean ± STD level of relative transcript abundance from three biological experiments. Asterisks * and ** indicate the significant difference between GmDGAT3-1 and GmDGAT3-2 at *p* < 0.05 and 0.01, respectively.

Furthermore, *GmDGAT3* expression profiling during soybean seed development displayed that *GmDGAT3-1* only had a low expression at the first three stages of soybean seed development (Figure 4A). However, *GmDGAT3-2* was highly expressed from 35 to 55 d, the period of fast increase of seed oil accumulation (Figure 4B). This data indicates that GmDGAT3-2 may play an important role in oil/TAG biosynthesis and accumulation in soybean seeds despite GmDGAT3-2 also possibly functioning in flowers and other tissues. Therefore, GmDGAT3-2 was selected for further investigation.

### Heterologous Expression of *GmDGAT3-2* Restores TAG Biosynthesis in the Yeast TAG-Deficient Mutant H1246

To determine whether GmDGAT3-2 has a high DGAT enzymatic activity, *GmDGAT3-2* was heterologously expressed in the TAG-deficient yeast mutant strain H1246. Yeast mutant H1246 lacks the four genes that encode, respectively, triacylglycerol biosynthetic enzymes, namely *DGA1* (member of the DGAT2 family), Lecithin cholesterol acyltransferase Related Open reading frame (*LRO1*, a diglyceride acyltransferase), and the Acyl-coenzyme A: cholesterol acyl transferase-Related Enzymes *ARE1* and *ARE2*. The H1246 strain thus produces neither TAGs nor oil bodies but is still viable, providing an excellent system to test the activity of enzymes such as DGAT or Phospholipid:diacylglycerol acyltransferase (PDAT) (Siloto et al., 2009). As a positive control, yeast *DGAT2* (*ScDGA1*) was also transferred into H1246. H1246 and the H1246 transformed with the empty vector pYES2.0 served as the negative control.

Nile Red was used to stain all genotypes of yeast cells after induction of transgene expression by galactose to detect TAGs and oil bodies (Supplementary Figure 4). Notably, a very tiny fluorescence signal occurred in the H1246 mutant transformed with the empty vector. However, a very strong fluorescence sign appeared in H1246 expressing *GmDGAT3-2*, suggesting that TAGs were formed in the *GmDGAT3-2-*transgenic yeast cells. And thus, GmDGAT3-2 restored TAG biosynthesis in yeast mutant H1246. It should be noticed that GmDGAT3-2 activity was not as high as ScDGA1 when overexpressed in this yeast mutant since the fluorescence signal was weaker in *GmDGAT3-2*-transgenic H1246 than that in H1246 transformed with *ScDGA1* (Supplementary Figure 4).

**Figure 5:**
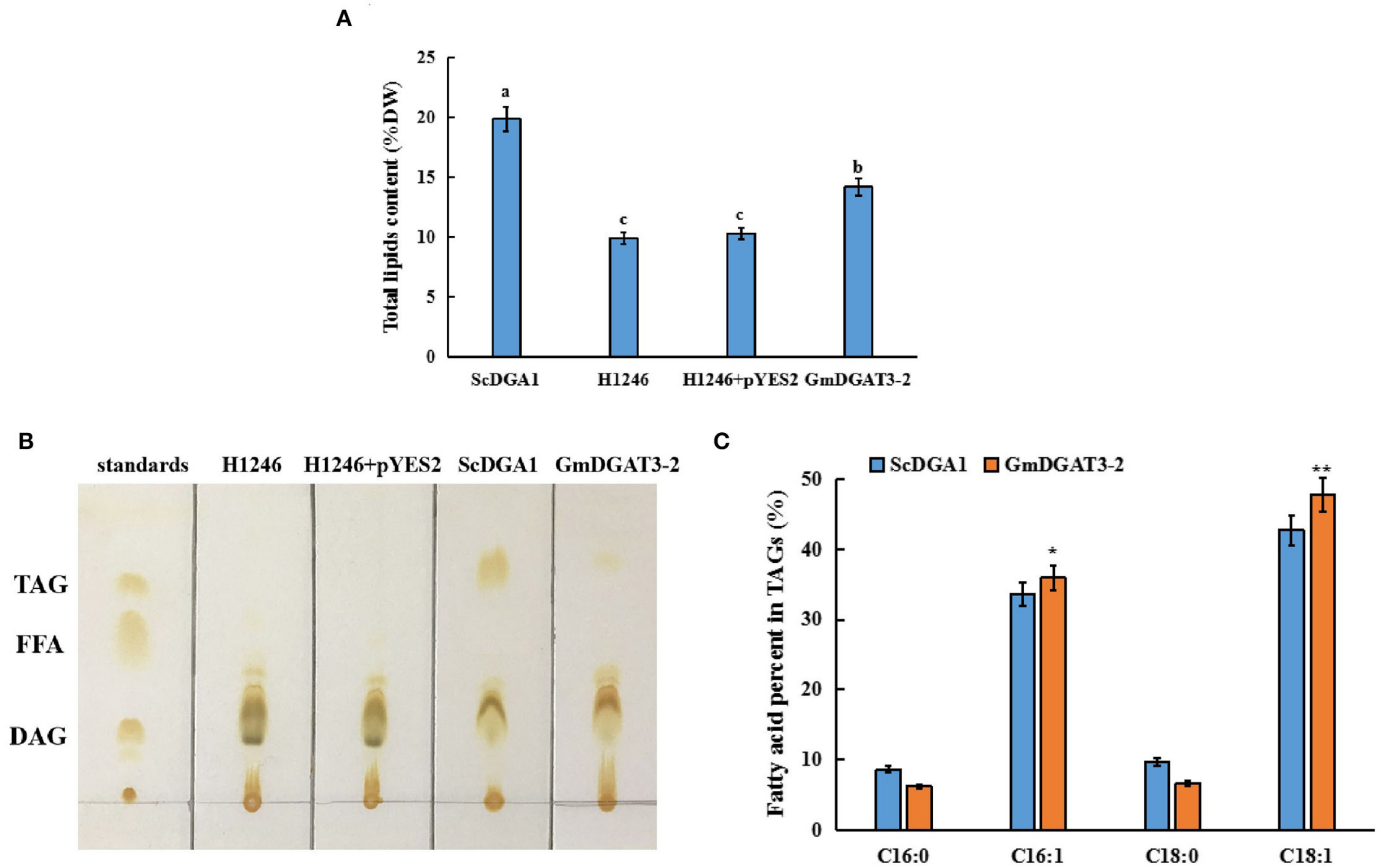
Lipid analysis in yeast H1246 transformed with *GmDGAT3-2* gene. **(A)** Total lipid content in yeast H1246 expressing *GmDGAT3-2*. The bars were *SD*s of three biological repeats. Different lowercase letters indicate significant differences at the *P* < 0.05 level. **(B)** Thin-layer chromatography (TLC) analysis of neutral lipids in yeast H1246 expressing *GmDGAT3-2* gene. Lipid spots were detected under UV after staining with iodine vapor. Lane 1, standards of acyl-lipid classes: TAG (triacylglycerol), FFA (free fatty acids), and DAG (diacylglycerols); Lane 2, H1246, the yeast strain H1246; Lane 3, H1246+pYES2, H1246 expressed empty vector (pYES2); Lane 4, ScDGA1, H1246 expressed yeast DGAT2; Lane 5, GmDGAT3-2, H1246 expressed GmDGAT3-2. **(C)** Fatty acid composition in TAG of yeast H1246 expressing *GmDGAT3-2* gene. ScDGA1, H1246 expressing yeast *DGAT2*. GmDGAT3-2, H1246 expressing soybean *GmDGAT3-2* gene. The bars were *SD*s of three biological repeats. Asterisks indicate significant difference between GmDGAT3-2 and the ScDGA1 according to *t*-test at **P < 0.01, and *P < 0.05, respectively.

A quantitative analysis of total lipid content was also conducted for those yeast cells. The yeast cells at the stationary stage were further cultured in the presence of galactose. Total lipids were extracted from the yeast samples for measurement. Total lipid content was low in the negative control, H1246 transformed with empty vector pYES2.0 whereas a high level of lipids was produced in H1246 transformed with *pYES2.0-ScDGA1*, the positive control (Figure 5A). Total lipid content in the H1246 transformed with *pYES2.0-GmDGAT3-2* increased by 32.8% over the negative control after induction of target gene expression by galactose. However, total lipid content in these transformants did not reach the same high levels as the positive control, suggesting that the activity of GmDGAT3-2 in yeast cells may be lower than that of ScDGA1, which is consistent with results revealed by the Nile Red staining assay described above. Thin-layer chromatography (TLC) was employed to separate total lipids in these yeast cells, showing that no TAG spot was detected in either the H1246 mutant or the H1246 transformed with the empty vector pYES2.0. By contrast, a TAG spot was presented in the yeast cells overexpressing the *GmDGAT3-2* gene despite this spot being weaker than that in *ScDGA1*-transgenic yeast (Figure 5B). Overall, these results evidence that GmDGAT3-2 has the DGAT enzymatic activity.

To determine fatty acid profiles in TAGs in the yeast transformed with *pYES2.0-GmDGAT3-2*, TAG spots were scraped off the TLC silica gel plate and then used to prepare FAMEs for analysis by gas chromatography. As shown in Figure 5C, the expression of GmDGAT3-2 in the H1246 mutant altered the fatty acid content in TAGs (% total fatty acids in TAGs), with a great increase in the unsaturated fatty acids C16:1 and C18:1 and a corresponding reduction in the saturated fatty acids C16:0 and C18:0. Moreover, levels of C16:1 and C18:1 were, respectively, 6.8 and 11.9% higher in the *GmDGAT3-2*-transgenic H1246 than that in the positive control expressing *ScDGA1*, suggesting that GmDGAT3-2 may have the substrate preference for monounsaturated fatty acids (MUFAs).

To verify this substrate specificity for MUFAs, particularly for 18:1, yeast feeding assays were conducted. Fatty acid supplementation experiments were performed by adding individual fatty acids at a concentration of 1 mM to the yeast culture medium. Since the main fatty acids in soybean seed oil include 18:2 (47.33%), 18:1 (15.66%), 18:3 (15.40%), and 16:0 (11.38%) (Jiang and Katuuramu, 2021), these four fatty acids plus 16:1 were used for yeast feeding assays, respectively.

The yeast growth curves were measured for yeast H1246 expressing *ScDGA1* or *GmDGAT3-2* when cultured with/without exogenous FA feeding (Supplementary Figure 5). The final OD600 value of the yeast culture was higher in the presence of exogenous FAs than that without feeding of exogenous FAs in the medium. Moreover, when fed with exogenous FAs, *GmDGAT3-2* transgenic yeast grew very fast, entering the logarithmic phase 10 h more ahead of the yeast expressing *ScDGA1*. The yeast biomass in the later culture period was significantly higher in the yeast cells expressing *GmDGAT3-2* fed with oleic acid than that in the yeast fed with other FAs (Supplementary Figure 5). These results revealed that when C18:1 was added to the medium, *GmDGAT3-2* transgenic H1246 yeast grew very fast and produced a larger amount of biomass.

TAG level was increased in H1246 yeast cells expressing *GmDGAT3-2* compared to the control cells expressing yeast *ScDGA1*, when exogenous 18:1 was added, demonstrating that GmDGAT3-2 could catalyze more TAG synthesis in the presence of exogenously added FAs, particularly when exogenous 18:1 was added. Importantly, a higher level of TAG synthesis catalyzed by GmDGAT3-2 in the transgenic yeast cells was achieved when cultured in the medium at the feeding of C18:1 compared to the feeding of other exogenous FAs (Supplementary Figure 6), indicating that GmDGAT3-2 might be selective for substrates containing C18:1.

Fatty acid profiling (Table 1) showed that for *ScDGA1*-transgenic H1246, compared to the control without exogenous FA addition, contents of 16:0, 16:1, 18:0, and 18:1 exhibited no significant changes when 18:2 and 18:3 were added, respectively, whereas levels of 18:1, 16:1, and 16:0 were just increased by 1–4%, respectively, at the presence of each of them separately in the medium. This indicated that ScDGA1 exhibited no significant difference in substrate specificity for 16:0, 16:1, and 18:1 except for no obvious selection for 18:2 and 18:3. However, when 18:3, 18:2, and 16:0 were separately supplied, the *GmDGAT3-2*-transgenic H1246 displayed that these three fatty acids increased by 2–6%, respectively, compared to the non-fed control yeast. Notably, contents of 18:1 and 16:1 were enhanced by 10.18 and 6.01% fold, respectively, in the *GmDGAT3-2*-transgenic yeast fed by 18:1 and 16:1 separately. These data demonstrated that GmDGAT3-2 had much higher substrate specificity for 18:1 than 16:1.

To further determine the enzymatic activity and substrate specificity of GmDGAT3-2, we conducted an *in vitro* enzyme activity characterization. A prokaryotic expression vector pET28 a^+^-*GmDGAT3-2* was constructed and then introduced into *E. coli* BL21. The positive BL21 cells bearing the *GmDGAT3-2* recombinant plasmid were activated and cultured in the liquid medium. When the culture mixture was up to OD600 = 0.3, isopropyl thiogalactoside (IPTG) with a concentration of 0.05 mmol·L^−1^ was added into the medium to induce high expression of GmDGAT3-2 for 6 h. The enzyme proteins were extracted and consequently used for enzyme activity assay *in vitro*.

**Figure 6:**
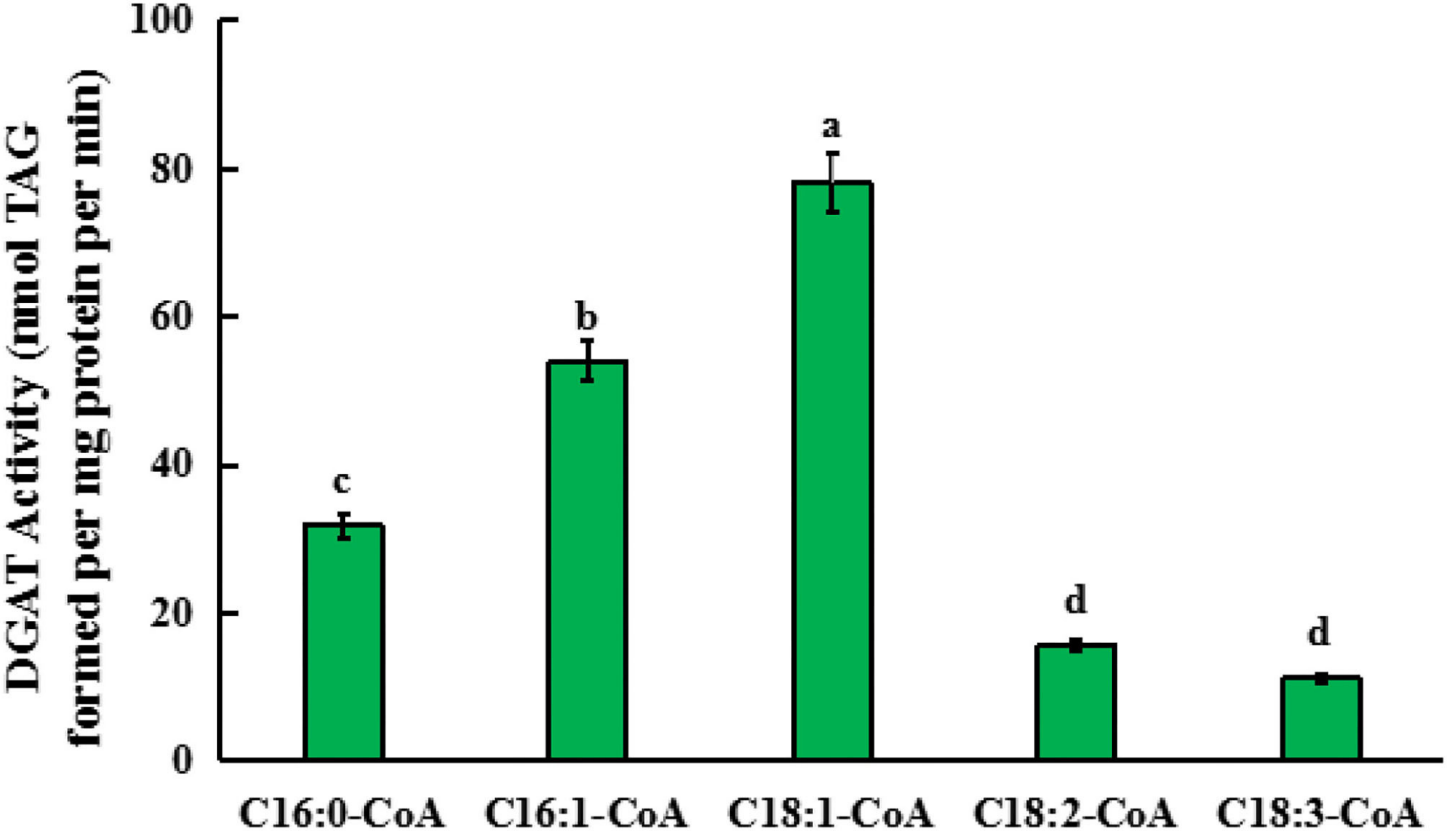
Quantification of the substrate specificity of GmDGAT3-2 for different acyl-CoAs by *in vitro* assay. Triglycerides (TAGs) formed when enzyme protein extracted from *E. coli* BL21 expressing GmDGAT3-2 (40 ng protein) were administered 5 μM of each acyl-CoA together with 100 μM of sn-1,2-DAG (18:1n9/16:0 DAG). Bar values are means ± SE (*n* = 3). Small letters (a and b) show significant difference (*P* < 0.05).

For this *in vitro* assay, 18:1n9/16:0 DAG (sn-1,2-diacylglycerol) was used as an acyl acceptor, and 18:3-CoA, 18:2-CoA, 18:1-CoA, 16:1-CoA, and 16:0-CoA were, respectively, added into the reaction system. After the reaction solution was incubated for 1 h, TAG content in the solution was detected to determine the activity of GmDGAT3-2 in the presence of each acyl-CoA. As shown in Figure 6, the TAG level in the presence of 18:1-CoA was much higher than that in the presence of 16:1-CoA while low enzymatic activity was observed when each of the other acyl-CoAs was added, respectively. This *in vitro* enzyme assay pieces of evidence that GmDGAT3-2 has strong substrate specificity for MUFAs, with much higher to 18:1 than 16:1.

### Transient Expression of *GmDGAT3-2* Leads to Oil Increase and Alteration in Fatty Acid Profiles in *Nicotiana benthamiana* Leaves

To further characterize the function of GmDGAT3-2, this gene was transiently introduced into *N. benthamiana* leaves by *Agrobacterium*-mediated infiltration. After 3 days of transfection, the infected leaf parts were collected to check the expression of *GmDGAT3-2* gene. As shown in Supplementary Figure 7, *GmDGAT3-2* was effectively expressed in *N. benthamiana* leaves infiltrated with the *pCAMBIA1303-GmDGAT3-2* construct, but not in leaves infiltrated with the empty vector, as revealed by RT-PCR.

**Figure 7:**
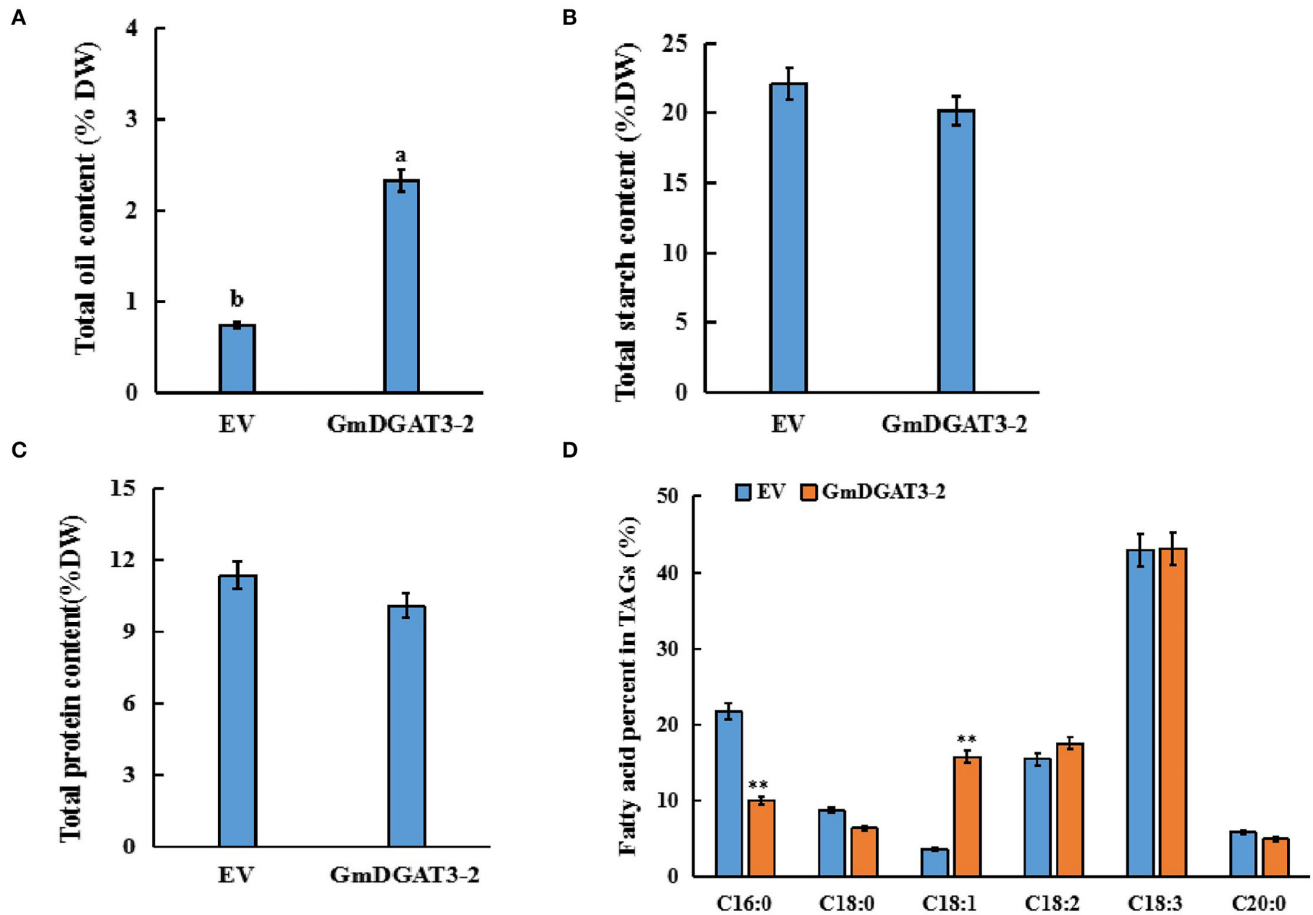
Contents of total oil **(A)**, protein **(B)**, starch **(C)**, and fatty acid profiles **(D)** in tobacco leaves expressing *GmDGAT3-2*. EV, tobacco leaves transiently expressing empty vector (pCAMBIA1303). GmDGAT3-2, tobacco leaves expressing *GmDGAT3-2*. The bars were *SD*s of three biological repeats. Lowercase letters indicate significant differences at the *P* < 0.05 level. Asterisks indicate significant difference from the EV according to *t*-test at **P < 0.01, and *P < 0.05, respectively.

After 5 days of transfection, the infected leaves were harvested for analysis of total oil, protein, and starch contents (Figure 7). Total oil content in the infiltrated leaves increased significantly by 1.6% compared to leaves infiltrated with the empty vector. Total protein and starch contents exhibited a slight decrease in the leaves infiltrated with the *pCAMBIA1303-GmDGAT3-2* construct although this difference was not significant (*P* < 0.05). These results suggested that heterogeneous overexpression of *GmDGAT3-2* could significantly improve the accumulation of oil in *N. benthamiana* leaves with a negligible effect on protein or starch contents.

To determine fatty acid profiles in the infected leaves, FAMEs prepared from the infiltrated tobacco leaves were examined by GC. As shown in Figure 7D, the transient overexpression of *GmDGAT3-2* significantly reduced the C16:0 content in the leaves by 11.7%, while C18:1 content was increased by 12.3% (*P* < 0.05). Changes in other fatty acids were not significant compared to the control. These results further suggested that GmDGAT3-2 had a high substrate specificity for oleic acid (C18:1) for TAG biosynthesis.

### Overexpression of *GmDGAT3-2* Significantly Increases Oil Accumulation With More Oleic Acid in the Transgenic Tobacco Seeds

**Figure 8:**
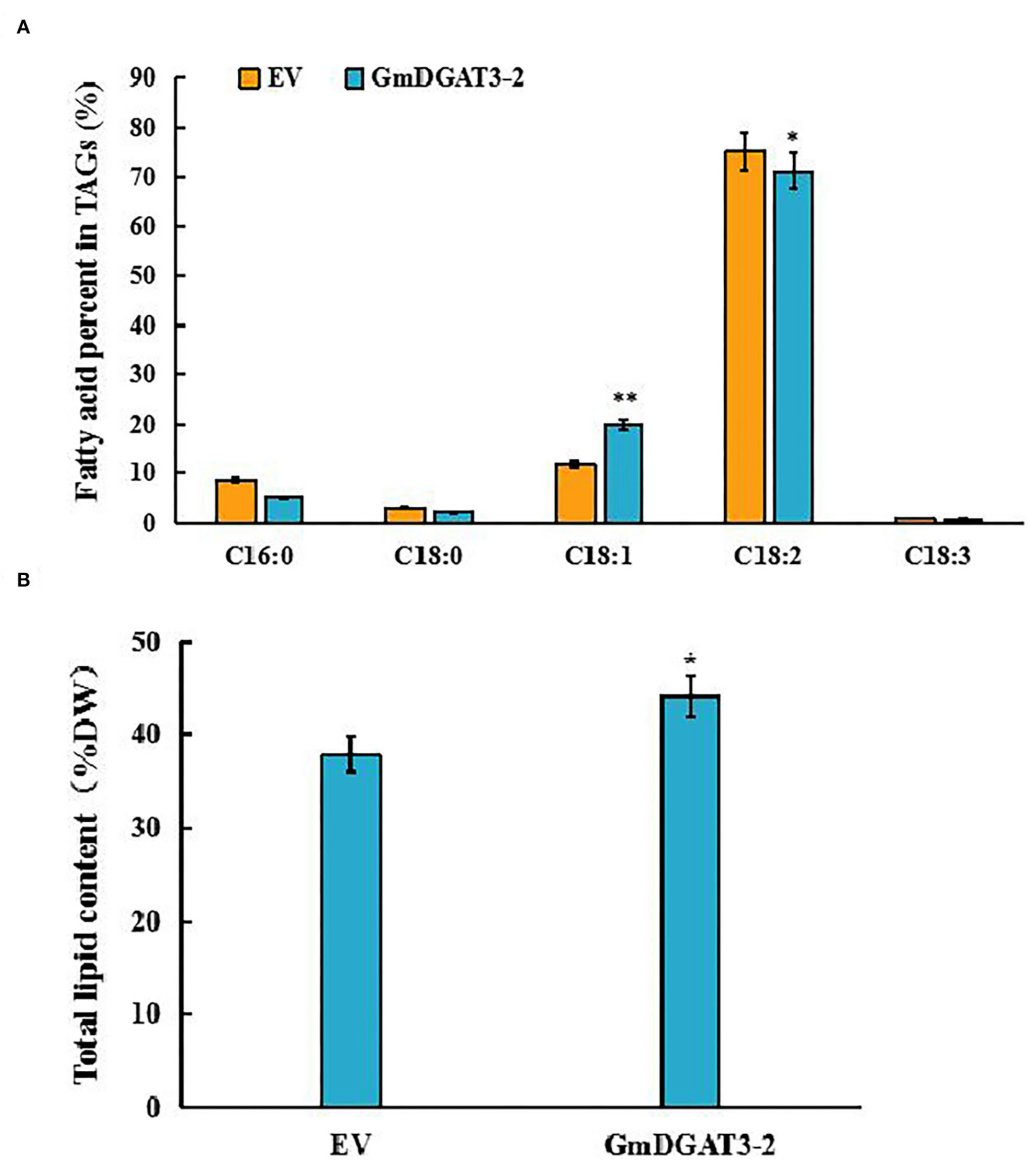
Major fatty acid profiles **(A)** and total lipid content **(B)** in tobacco seeds of transgenic tobacco expressing *GmDGAT3-2* and empty vector, respectively. GmDGAT3-2, GmDGAT3-2 transgenic tobacco plants; EV, empty vector transformed tobacco plants. The bars were means and *SD*s of three biological repeats. Asterisks indicate significant difference from the EV according to *t*-test at **P < 0.01, and *P < 0.05, respectively.

To investigate whether *GmDGAT3*-2 overexpression leads to seed oil increase and alteration of fatty acid composition, the transgenic tobacco seeds were harvested for lipid analysis. The data of seed oil content and fatty acid profiles in the *GmDGAT3*-2 transgenic tobacco (Figure 8) showed that heterogeneous expression of *GmDGAT3*-2 resulted in seed oil enhancement by 16.6% compared to the EV-transformed tobacco plants. Seed fatty acid profiling demonstrated that the oleic acid (18:1) level was increased by 1.68-fold in the transgenic tobacco plants compared with the control. The transgenic tobacco seed data also displayed that GmDGAT3-2 has the high enzymatic activity of TAG and substrate specificity for oleic acid, suggesting that GmDGAT3-2 may function importantly in soybean seed oil accumulation.

## Discussion

To meet the growing demand for vegetable oils for applications in human food, animal feed, biodiesel, and oleochemical industries, it is of significance to increase vegetable oil yields and quality. Dissecting the mechanism underlying lipid biosynthesis and regulation in oilseeds can provide the scientific basis for oil-crop genetic improvements. DGAT enzyme is the key target in this regard (Ichihara et al., 1988; Perry et al., 1999; Jako et al., 2001; He et al., 2004; Sørensen et al., 2005; Lung and Weselake, 2006). Compared to well-characterized DGAT1s and DGAT2s, DGAT3s were not functionally analyzed in many plants. To date, one or two DGAT3 members were detected in several plant species. For example, one DGAT3 member was examined in purple false brome (*Brachypodium dystachion*), maize (*Zea mays*), and rice (*Oryza sativa*). Two DGAT3 members were identified in banana (*Musa acuminata*), cotton (*Gossipium hirsutum*), African oil palm (*Elaeis guineensis*), and rapeseed (Turchetto-Zolet et al., 2016; Rosli et al., 2018). In this study, we identified two DGAT3 members from the soybean genome, denoted as GmDGAT3-1 and GmDGAT3-2 (Figures 1, 2 and Supplementary Table 2). The presence of two DGAT3 members may stem from the genome duplication events, just like the case for most genes in the allotetraploid soybean genome. Gene duplications may provide the opportunity for genes to evolve new functions without compromising essential biological functions, a process known as sub-functionalization (Qian and Zhang, 2014).

An *in silico* physicochemical analysis showed that GmDGAT3-1 and GmDGAT3-2 proteins were cytosolic and soluble proteins (Supplementary Figure 1 and Supplementary Table 3), which are different from soybean GmDGAT1s and GmDGAT2s that are hydrophobic and localized in the endoplasmic reticulum. Consistence with our findings, previous reports also indicated that DGAT3 proteins are soluble and cytosolic in a few plant species tested (Saha et al., 2006; Hernandez et al., 2012; Turchetto-Zolet et al., 2016). Our data showed that soybean DGAT3 contained a TRX-like Fd domain, similar to Arabidopsis and peanut DGAT3s (Aymé et al., 2018). Multiple sequence alignment analysis (Figure 1) further demonstrated that GmDGAT3-1 and GmDGAT3-2 both contained several additional functional domains typically conserved in DGAT3 proteins, such as acyltransferase binding sites which determine the substrate specificity of the enzyme. Finally, phylogenetic analysis (Figure 2) revealed that GmDGAT3s shares high similarity with mung bean DGAT3, and possibly have a similar DGAT enzymatic activity.

The expression pattern of a given gene in the multiple-tissue organism can provide information for its functions. *GmDGAT3-1* and *GmDGAT3-2* transcript levels were examined across multiple soybean tissues in this study (Figure 3). *GmDGAT3-2* (Glyma13g17860) was more highly expressed in all tissues tested than *GmDGAT3-1* (Glyma17g04650), with the highest abundance in flowers and higher level in stems and seeds. Such different expressions for two isoforms of *DGAT3*s were also observed in African oil palm where *EgDGAT3-1* showed high expression in early fruits, and *EgDGAT3-2* expressed in the mesocarp (Rosli et al., 2018). Thus, different *DGAT3* genes within the same species may display distinct expression patterns in different tissues and possible differentiated functions. Our data on *GmDGAT3-1* and *GmDGAT3-2* expressions during soybean seed development (Figure 4) further exhibited that the *GmDGAT3-2* expression pattern was consistent with the fast increase of seed oil accumulation, indicating that *GmDGAT3*-2 may contribute importantly to oil biosynthesis and accumulation in soybean seeds.

The functional complementary assay by heterologously expressing *GmDGAT3-2* in yeast TAG-deficient mutant H1246 revealed that GmDGAT3-2 can restore TAG synthesis, and thus have a DGAT enzymatic activity (Figure 5 and Supplementary Figure 4) with a possible substrate preference for 18:1 and 16:1. Similarly, two peanut DGAT3s, AhDGAT3-2, and AhDGAT3-3 were identified to have such DGAT activity when expressed in the yeast (Chi et al., 2014). Soluble proteins with DGAT activity had also been characterized by the diatom *Phaeodactylum tricornutum* (Cui et al., 2013), the yeast *Rhodotorula glutinis* (Rani et al., 2013), and the alga *Chlamydomonas reinhardtii* (Bagnato et al., 2017). Arabidopsis DGAT3 was detected to act in germinating seedlings, involved in the recycling of C18:2 and C18:3 into TAGs (Aymé et al., 2018). Together, DGAT3 is thought to contribute importantly to another unique pathway of TAG biosynthesis (Saha et al., 2006; Hernandez et al., 2012). To verify the substrate specificity of GmDGAT3-2, yeast feeding assays and *in vitro* enzyme activity characterization were also carried out (Table 1, Figure 6, and Supplementary Figure 6), again showing that GmDGAT3-2 has high substrate specificity for oleic acid (18:1).

Further functional characterization was conducted to overexpress *GmDGAT3-2* in tobacco leaves. Our results (Figure 7) showed that total oil content was significantly increased in *N. benthamiana* leaves overexpressing *GmDGAT3-2*, accompanied by a slight decrease in protein and starch content. It was reported that soybean seeds specifically expressing *UrDGAT2A* from the fungus *Umbelopsis ramanniana* had a 1.5% oil increase but a considerable reduction in protein content (Lardizabal et al., 2008). Higher levels of C18:1 (Figure 7) were also achieved in the tobacco leaves expressing *GmDGAT3-2*. Moreover, lipid analysis in the transgenic tobacco seeds (Figure 8) demonstrated that heterogeneous expression of *GmDGAT3*-2 significantly increased seed oil content, with a much high level of oleic acid. These results evidence that GmDGAT3-2 has a high DGAT enzymatic activity and substrate preference for MUFAs, particularly oleic acid, indicating that GmDGAT3-2 may function importantly in soybean seed oil accumulation.

Previous studies on functions of DGAT3 detected that some DGAT3 members exhibited substrate preference. For example, a CsDGAT3-3 was identified from *Camelina sativa* seeds enriched with UFA such as ALA, LA, and EA (20:1), and this CsDGAT3-3 showed a high substrate preference for C20:1 (Gao et al., 2021). The overexpression *Phaeodactylum tricornutum PtDGAT3* gene resulted in the increased incorporation of unsaturated C18 fatty acids into TAGs (Rani et al., 2013), indicating that PtDGAT3 had substrate specificity to unsaturated C18 fatty acids. Cytosolic AtDGAT3 was identified to function in the recycling of 18:3 to TAG *via* regulating the acyl-CoA pool size and composition in response to the needs of membrane lipid biosynthesis in young seedlings (Hernandez et al., 2012). In the present study, our analysis by combining feeding assays and *in vivo* enzyme activity characterization (Figure 6, Supplementary Figure 6, and Table 1), as well as tobacco transient expression (Figure 7) and stable transformation (Figure 8), revealed that GmDGAT3-2 has substrate selection for UFAs, particularly for C18:1, one of the main FAs (~21%) in soybean oil. Such substrate preference of the DGAT3 enzyme may be related to the abundance of available fatty acid substrate or the 3D architecture of different DGAT3 proteins. Collectively, DGAT3 members derived from different plant species may perform different functions. The mechanism for such diverse functions needs further investigation in detail. And GmDGAT3-2 has a substrate preference for 18:1, unlike AtDGAT which has a substrate preference for 18:3. Our oncoming work is to investigate whether GmDGAT3-2 play a role in seed germinating and seedling growth of soybean. Moreover, whether GmDGAT3-2 has a substrate preference for very short or very-long-chain acyl-CoAs will be examined in our future study.

## Conclusion

In conclusion, we characterized two *DGAT3* genes from soybean that encode putative soluble and cytosolic enzyme proteins containing the typical TRX-like Fd domain responsible for binding a [2Fe-2S] cluster. The *GmDGAT3* genes exhibited different expression patterns in various soybean tissues, with *GmDGAT3-2* showing higher expression than *GmDGAT3-1* in all tissues tested, indicating that GmDGAt3-2 may function much important for TAG biosynthesis in seed development. Functional characterization by yeast complementation assay, feeding test with exogenous FAs, *in vitro* enzyme activity experiment, and tobacco genetic transformation evidence that GmDGAT3-2 has strong DGAT enzymatic activity and higher substrate preference for MUFAs, especially oleic acid (C18:1). This study provides new information for understanding an alternate pathway of TAG biosynthesis in soybean seeds, showing that GmDGAT3-2 can be an excellent molecular target for genetic improvement of soybean or other oilseeds to increase oil yield and quality.

## Data Availability Statement

The data presented in the study are deposited in the DRYAD repository, accession number 10.3389/fpls.2022.854103.

## Author Contributions

RL, JX, and XJ conceived and designed the experiments. JX, HG, YX, RS, ML, LH, YG, YZ, FZ, and HZ performed experiments and analyzed the data. JX and HG wrote the manuscript. RL and XJ revised the manuscript. All authors read and approved the final manuscript.

## Funding

The research was supported by the National Natural Science Foundation of China (31401430), the Key Research and Development Program of Shanxi Province (201703D221002-3), Colleges and Universities Scientific Research Outstanding Achievement Cultivation Project of Shanxi Province, the Training Project of Research Achievements of Universities in Shanxi Province, Science and Technology Innovation of Higher Education of Shanxi Province (2021L112), Biological Breeding Engineering of Shanxi Agricultural University (YZGC101), Basic Research Program of Shanxi Province (20210302124170), 2021 Graduate Innovation Project of Shanxi Province, China (No. 2021Y310), Applied Basic Research Program of Shanxi Academy of Agricultural Science (YGC2019FZ4), and Local Science and Technology Development Project Guided by the Central Government (2020).

## Conflict of Interest

The authors declare that the research was conducted in the absence of any commercial or financial relationships that could be construed as a potential conflict of interest.

## Publisher's Note

All claims expressed in this article are solely those of the authors and do not necessarily represent those of their affiliated organizations, or those of the publisher, the editors and the reviewers. Any product that may be evaluated in this article, or claim that may be made by its manufacturer, is not guaranteed or endorsed by the publisher.
