# Supplementary material for: Functional Characterization of Soybean Diacylglycerol Acyltransferase 3 in Yeast and Soybean
Source: Front Plant Sci. 2022 May 25;13:854103. doi: 10.3389/fpls.2022.854103 (PMC9174931; doi:10.3389/fpls.2022.854103)
Supplement: Supplementary file 1 [file Data_Sheet_1.PDF]

Supplementary Material

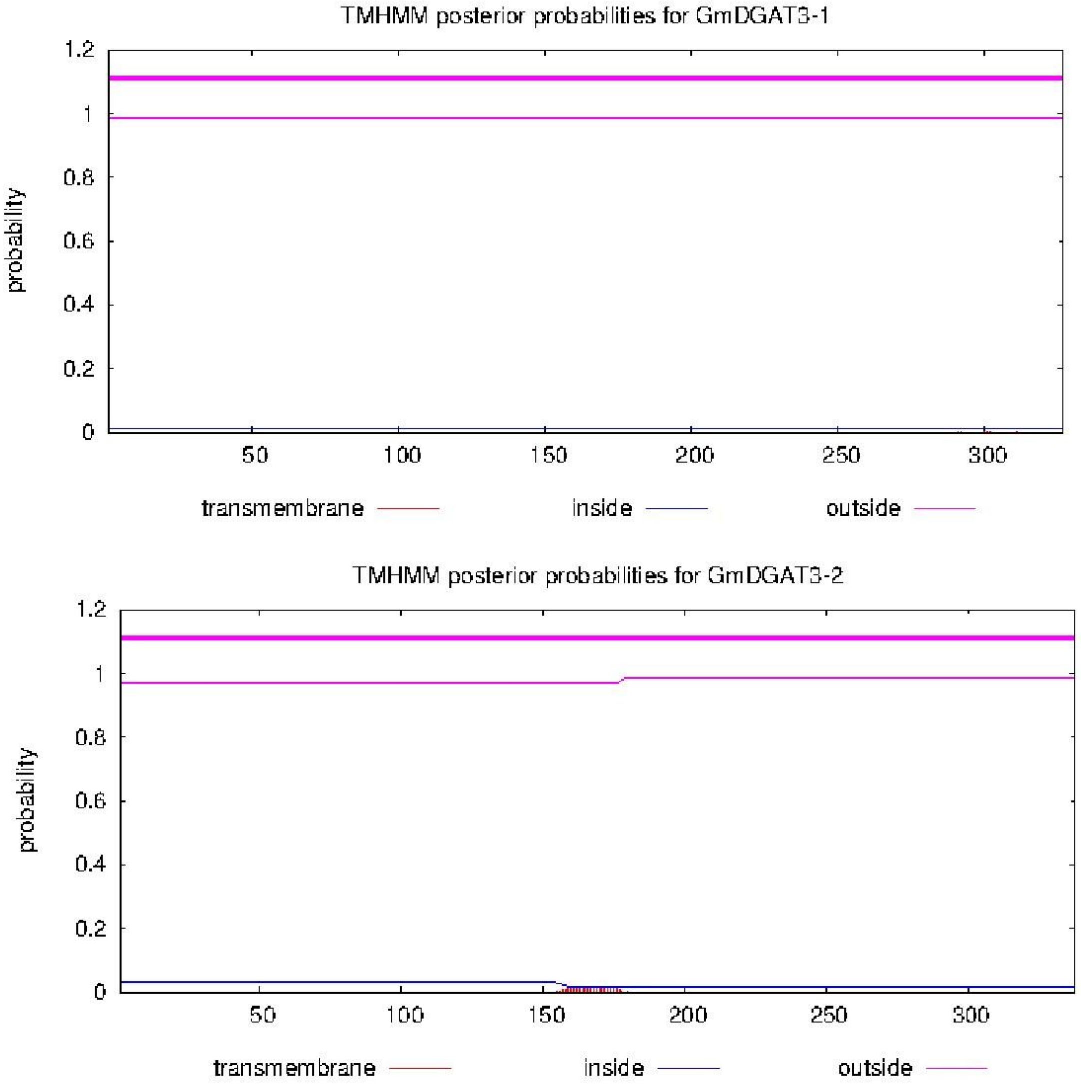

Fig. S1 Predicted transmembrane domain for soybean GmDGAT3-1 and GmDGAT3-2 protein sequences.

The transmembrane regions are shown in red, while regions of all sequences predicted to be located inside or outside the membrane are shown in blue and pink, respectively. No transmembrane region was found for GmDGAT3-1 and GmDGAT3-2 protein.

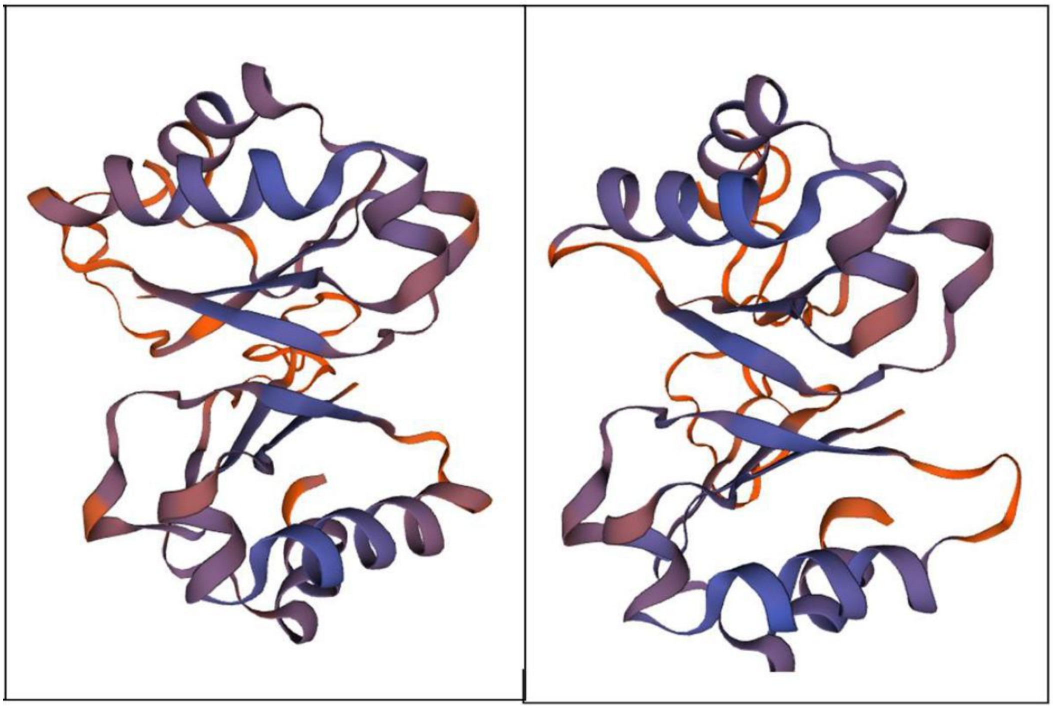

GmDGAT3-1 GmDGAT3-2

Fig. S2 Three-dimensional structure prediction of GmDGAT3 proteins

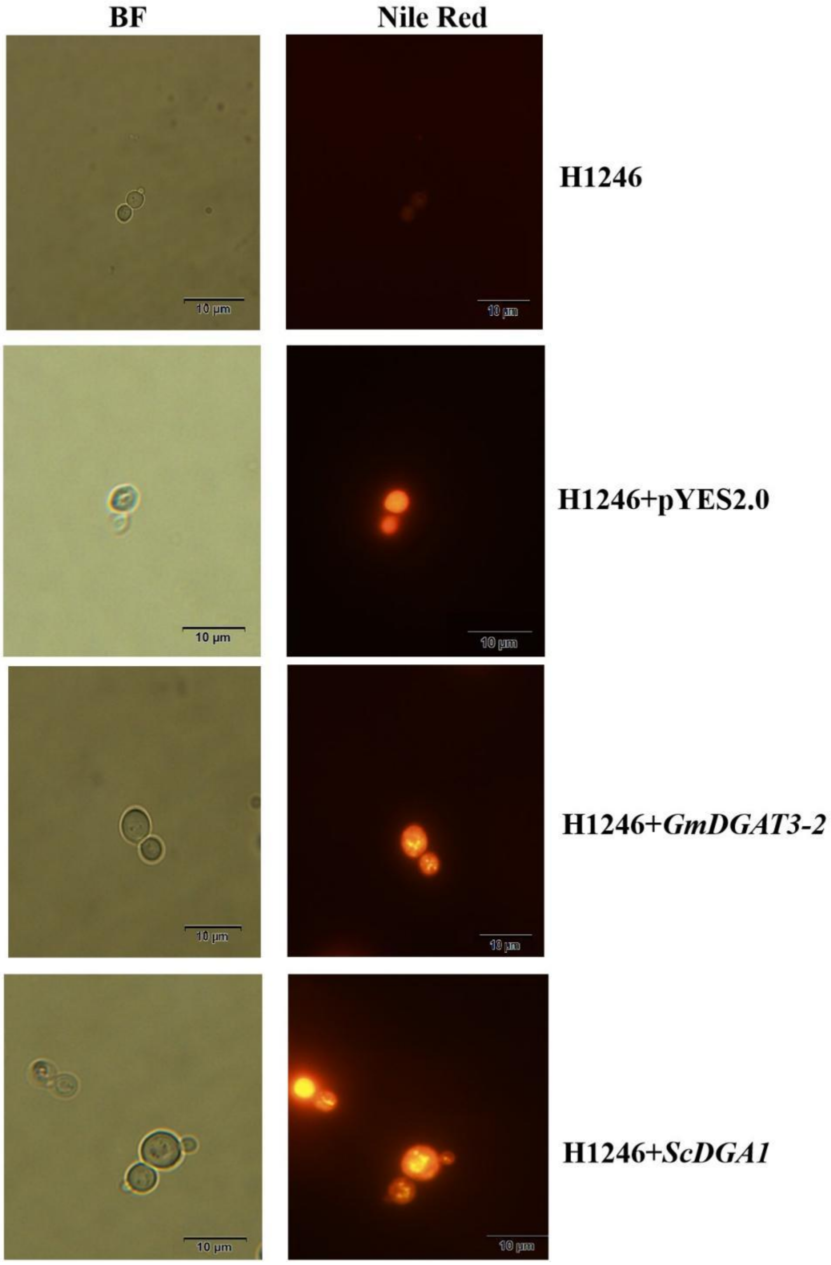

Fig. S3 Nile Red staining of the yeast H1246 cells expressing *GmDGAT3-2*

The lipid bodies' formation and accumulation in yeast cell were stained with Nile Red and visualized with fluorescence microscope of PH50TV. H1246, TAG-deficient mutant strain H1246; H1246+pYES2 (negative control), H1246 harboring the empty vector (pYES2); H1246+GmDGAT3-2, H1246 expressing soybean *GmDGAT3-2* gene; H1246+ScDGA1, H1246 expressing yeast DGAT2 (positive control). BF, the images were visualized under optical microscope. Nile Red, the images were visualized under fluorescence microscope.

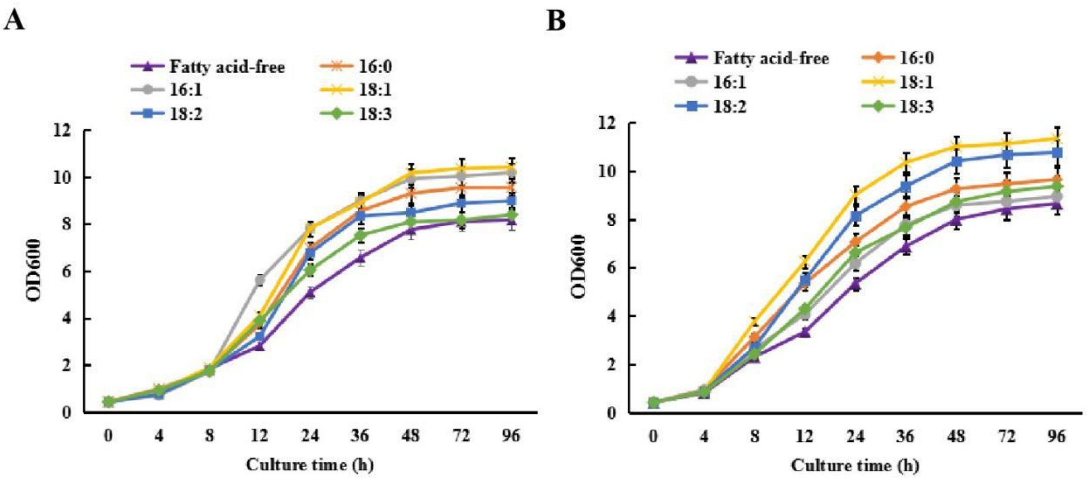

Fig. S4 Growth curve of the yeast H1246 cultivated in the synthetic minimal medium added with each of exogenous fatty acid at a final concentration of 1 mM.

A, H1234 yeast cells expressing *ScDGA1*. B, H1246 yeast cells expressing *GmDGAT3-2*. Data are presented as mean ± SD from triple biological replicates.

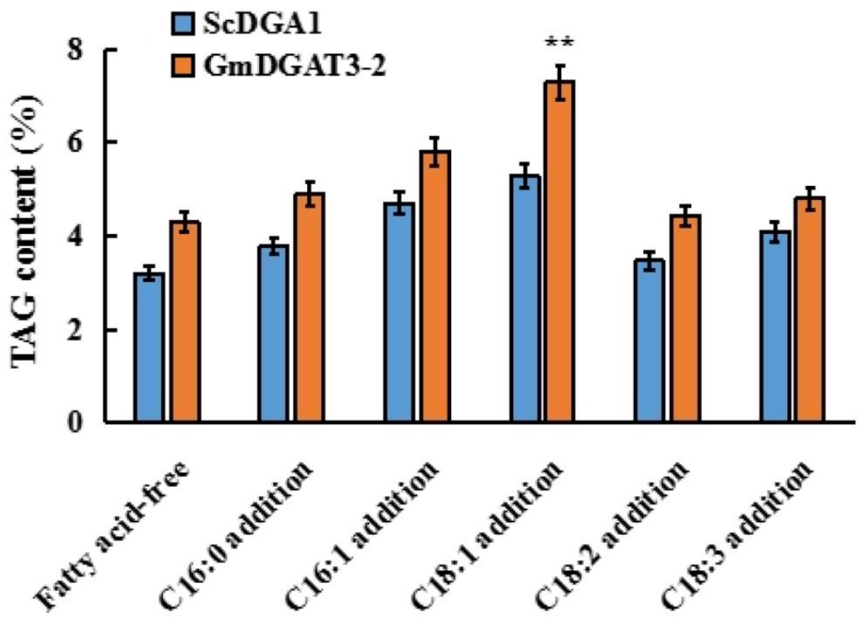

Fig. S5 TAG content in H1246 yeast cells expressing *ScDGA1* or *GmDGAT3-2* in the medium with and without adding of exogenous fatty acids.

Bar represented mean ± SE (n=3) for the yeast cells cultured at presence or absence of exogenous fatty acids. Asterisks indicate significant difference from the wild type according to t-test at ** $P < 0.01$, and * $P < 0.05$, respectively.

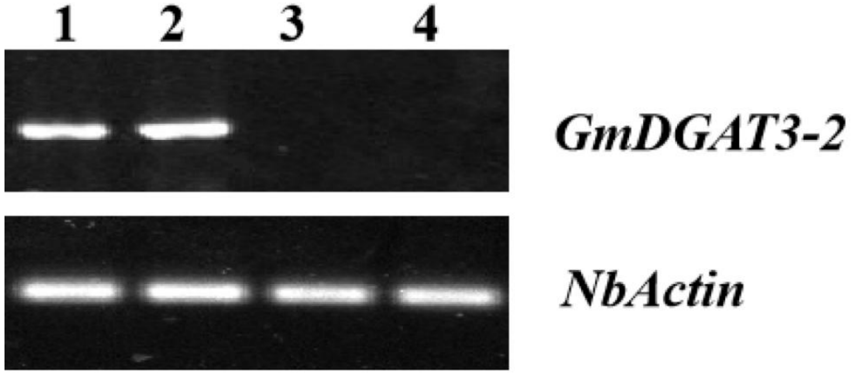

Fig. S6 Gel image of RT-qPCR for *GmDGAT3-2* expression.

The total RNA from tobacco leaves at 5 days of infection was used for cDNA synthesis. The target transcripts were obtained after 25 cycles using cDNA as template. The *NbActin* gene was used as positive control. Transient expressing empty vector (pCAMBIA1303) (the last two lanes) in tobacco leaves was used as the negative control. The lane 1 and 2 were the tobacco leaves expressing GmDGAT3-2.

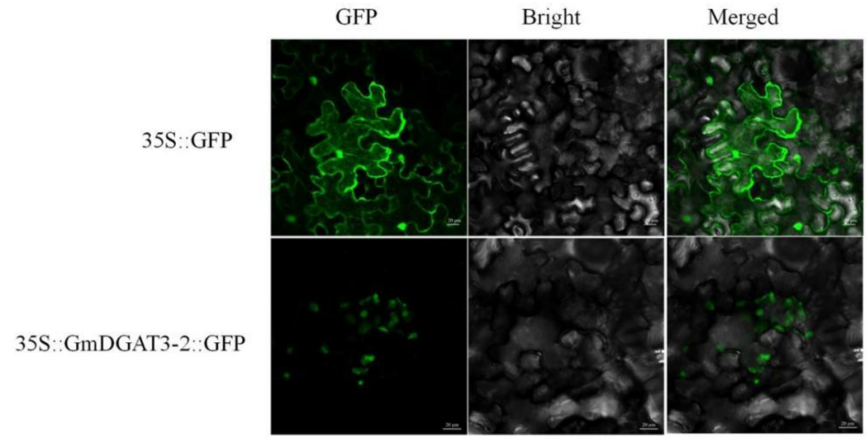

Fig. S7 Subcellular localization of the GmDGAT3-2-GFP fusion proteins by transient expression in tobacco and examined using fluorescence microscopy.

Table S1 Primer sequences used for this article

Primers	Sequence（5'-3'）
GmDGAT3-1-qPCR-F	TGGCGTGGCTTCTCGTGTT
GmDGAT3-1-qPCR-R	ACATGGCAAGTTTCTCGGGATC
GmDGAT3-2-qPCR-F	ACCTCGGATGCTGGAGATGTAT
GmDGAT3-2-qPCR-R	AACCACAATCGCTGTCACTTGA
GmActin-qPCR-F	AAGCTGTTCTCTCCTTGTACGCC
GmActin-qPCR-R	GCACAGTGTGAGACACACCATCA
GmDGAT3-2-CDs-F	ATGGAGATCTCCGGCACC
GmDGAT3-2-CDs-R	TCAAGTAGCTGTAGCTGCACCT
M13-F	GTAAAACGACGGCCAGT
M13-R	CAGGAAACAGCTATGAC
GmDGAT3-2-pYES2-F	C<u>GAGCTC</u>ATGGAGATCTCCGGCACC
GmDGAT3-2-pYES2-R	AT<u>GCGGCCGC</u>TCAAGTAGCTGTAGCTGCACCT
pYES2-F	TAATACGACTCACTATAGGG
pYES2-R	GTAGTACATTAATCAATACAGTG
GmDGAT3-2-1303-F	GC<u>TCTAGA</u>ATGGAGATCTCCGGCACC
GmDGAT3-2-1303-R	CC<u>TTAATTAA</u>TCAAGTAGCTGTAGCTGCACCT
NbActin-F	CAGTGGCCGTACAACAGGTA
NbActin-R	AACCGAAGAATTGCATGAGG

Table S2 Basic information of DGAT proteins from different plant specie

Latin name	Protein	NCBI accession number
Arabidopsis thaliana	AtDGAT1	AT2G19450
	AtDGAT2	AT3G51520
	AtDGAT3	OAP16619.1
Arachis hypogaea	AhDGAT3	AAX62735.1
Brassica napus	BnDGAT3	XP_013655610.1
Camelina sativa	CsDGAT3	AQM52372.1
Capsella rubella	CrDGAT3	XP_006304457.1
Eutrema salsugineum	EsDGAT3	XP_006393443.1
Glycine max	GmDGAT1	Glyma13G106100.1
	GmDGAT1	Glyma17G053300.1
	GmDGAT1	Glyma9G065300.1
	GmDGAT2	Glyma9G195400.1
	GmDGAT2	Glyma16G115800.1
	GmDGAT2	Glyma16G115700.1
	GmDGAT2	Glyma1G156000.1
	GmDGAT2	Glyma11G088800.1
	GmDGAT3-1	Glyma.13G118300.1
	GmDGAT3-2	Glyma.17G041600.1
Populus trichocarpa	PtDGAT3	XP_002314335.2
Vigna radiata var. radiata	VrDGAT3	XP_014505613.1

Table S3 Physicochemical properties of GmDGAT3 proteins in soybean

Protein	Protein Length (aa)	Molecular Weight （kD）	Theoretical pI	Formula	Instability Index	Hydrophility
AhDGAT3-1	345	37.75	9.12	$C_{1611}H_{2665}N_{481}O_{511}S_{25}$	46.04	-0.470
GmDGAT3-1	327	34.73	8.52	$C_{1481}H_{2459}N_{433}O_{479}S_{23}$	40.63	-0.263
GmDGAT3-2	338	35.88	8.61	$C_{1547}H_{2528}N_{442}O_{489}S_{23}$	38.95	-0.227
