# Supplementary material for: Functional Characterization of Soybean Diacylglycerol Acyltransferase 3 in Yeast and Soybean
Source: Front Plant Sci. 2022 May 25;13:854103. doi: 10.3389/fpls.2022.854103 (PMC9174931; doi:10.3389/fpls.2022.854103)
Supplement: Supplementary file 2 [file Table_1.docx]

**Supplementary data**

**Supplementary Figure 1 Predicted transmembrane domain for soybean GmDGAT3-1 and GmDGAT3-2 protein sequences.**

The transmembrane regions are shown in red, while regions of all sequences predicted to be located inside or outside the membrane are shown in blue and pink, respectively. No transmembrane region was found for GmDGAT3-1 and GmDGAT3-2 protein.

**Supplementary Figure 2 Three-dimensional structure prediction of GmDGAT3 proteins**

**Supplementary Figure 3 Subcellular localization of the GmDGAT3-2-GFP fusion proteins by transient expression in tobacco and examined by fluorescence microscopy.**

**Supplementary Figure 4 Nile Red staining of the yeast H1246 cells expressing *GmDGAT3-2***

The lipid bodies’ formation and accumulation in yeast cell were stained with Nile Red and visualized with fluorescence microscope of PH50TV. H1246, TAG-deficient mutant strain H1246; H1246+pYES2 (negative control), H1246 harboring the empty vector (pYES2); H1246+GmDGAT3-2, H1246 expressing soybean *GmDGAT3-2* gene; H1246+ScDGA1, H1246 expressing yeast *DGAT2* (positive control). BF, the images were visualized under optical microscope. Nile Red, the images were visualized under fluorescence microscope.

**Supplementary Figure 5 Growth curve of the yeast H1246 cultivated in the synthetic minimal medium added with each of exogenous fatty acid at a final concentration of 1 mM.**

A, H1246 yeast cells expressing *ScDGA1*. B, H1246 yeast cells expressing *GmDGAT3-2*. Data are presented as mean ± SD from triple biological replicates.

**Supplementary Figure 6 % TAG of total lipids in H1246 yeast cells expressing *ScDGA1* or *GmDGAT3-2* in the medium with and without adding of exogenous fatty acids.**

Bar represented mean ± SE (n=3) for the yeast cells cultured at presence or absence of exogenous fatty acids. Asterisks indicate significant difference from the wild type according to *t*-test at ** *P* < 0.01, and * *P* < 0.05, respectively.

**Supplementary Figure 7 Gel image of semi-quantitative PCR for *GmDGAT3-2* expression.**

The total RNA from tobacco leaves at 5 days of infection was used for cDNA synthesis. The target transcripts were obtained after 25 cycles using cDNA as template. The *NbActin* gene was used as the internal control. Transient expressing empty vector (pCAMBIA1303) (the last two lanes) in tobacco leaves was used as the negative control. The lane 1 and 2 were the tobacco leaves expressing *GmDGAT3-2*.

**Supplementary Table 1 Primer sequences used for this article.**

**Supplementary Table 2 Basic information of DGAT proteins from different plant species.**

**Supplementary Table 3 Physicochemical properties of GmDGAT3 proteins in soybean.**
